# Global Tree Cover and Biomass Carbon on Agricultural Land: The contribution of agroforestry to global and national carbon budgets

**DOI:** 10.1038/srep29987

**Published:** 2016-07-20

**Authors:** Robert J. Zomer, Henry Neufeldt, Jianchu Xu, Antje Ahrends, Deborah Bossio, Antonio Trabucco, Meine van Noordwijk, Mingcheng Wang

**Affiliations:** 1 Key Laboratory for Plant Diversity and Biogeography of East Asia (KLPB), Kunming Institute of Botany, Chinese Academy of Science, Kunming 650201, Yunnan, China; 2 Centre for Mountain Ecosystem Studies, World Agroforestry Center (ICRAF), East and Central Asia Region, Kunming 650201, China; 3 World Agroforestry Centre (ICRAF), Nairobi, Kenya; 4 Royal Botanic Garden Edinburgh, 20A Inverleith Row, EH3 5LR, Edinburgh, UK; 5 International Center for Tropical Agriculture (CIAT), Soils Division, Nairobi, Kenya; 6 Euro-Mediterranean Center on Climate Change, IAFES Division, Sassari, Italy; 7 Department of Science for Nature and Environmental Resources (DIPNET), University of Sassari, Via De Nicola 9, 07100 Sassari, Italy; 8 World Agroforestry Center (ICRAF) – Southeast Asia Regional Office, Bogor, Indonesia; 9 Wageningen University and Research, Plant Production Systems, Wageningen, the Netherlands

## Abstract

Agroforestry systems and tree cover on agricultural land make an important contribution to climate change mitigation, but are not systematically accounted for in either global carbon budgets or national carbon accounting. This paper assesses the role of trees on agricultural land and their significance for carbon sequestration at a global level, along with recent change trends. Remote sensing data show that in 2010, 43% of all agricultural land globally had at least 10% tree cover and that this has increased by 2% over the previous ten years. Combining geographically and bioclimatically stratified Intergovernmental Panel on Climate Change (IPCC) Tier 1 default estimates of carbon storage with this tree cover analysis, we estimated 45.3 PgC on agricultural land globally, with trees contributing >75%. Between 2000 and 2010 tree cover increased by 3.7%, resulting in an increase of >2 PgC (or 4.6%) of biomass carbon. On average, globally, biomass carbon increased from 20.4 to 21.4 tC ha^−1^. Regional and country-level variation in stocks and trends were mapped and tabulated globally, and for all countries. Brazil, Indonesia, China and India had the largest increases in biomass carbon stored on agricultural land, while Argentina, Myanmar, and Sierra Leone had the largest decreases.

Global carbon, water and nutrient cycles have all been profoundly impacted by the historical and ongoing increase of agricultural production worldwide^1^,^2^,^3^,^4^. Both land use change to agriculture and agricultural production have contributed, and continue to contribute, significantly to the projected impacts of global climatic warming^5^, with notable implications for food security^6^,^7^,^8^. Within this context, tree cover on agricultural land has the potential to make an important contribution to climate change mitigation^9^,^10^,^11^,^12^. The global role of tree-based carbon sequestration on agricultural land is thus far poorly understood and possibly has been significantly underestimated. Agricultural production and ongoing land use change contribute significantly to greenhouse gas (GHG) emissions, accounting for 24% globally^13^. Almost 50% of all potentially vegetated land surface globally has been converted to croplands, pastures and rangelands^1^,^3^,^4^, and these continue to expand to feed the planet’s growing population^8^. Today most of this expansion is taking place in the tropics where an estimated 80% of this expansion is replacing forests^14^. Within this context, there is an increasing global recognition of the need for incentives for agricultural practices that reduce carbon emissions from both crop and livestock production^12^. Recent negotiations within UN Framework Convention on Climate Change (UNFCCC) have considered a broader land use agreement, combining the proposed mechanisms for ‘Reducing Emissions from Deforestation and Forest Degradation in Developing Countries’ (REDD+) with incentives to reduce GHG emissions from agriculture^15^.

Agroforestry - a diversified set of agricultural production systems that integrate trees in the agricultural landscape - is often discussed in this regard as a strategy that can be used both for adaptation and mitigation. It is extensively practiced throughout tropical and developing countries, with an estimated 1.2 billion people around the world dependent upon agroforestry farming systems^16^. While the importance of biomass carbon in forests (above and below ground) is widely recognized^17^,^18^,^19^, the biomass carbon pool on agricultural land is seen as arguably negligible compared to the soil organic carbon (SOC) pool^20^,^21^. However, given the vast scale of available agricultural land, estimates of tree cover on agricultural land globally^22^,^23^, and the role of woody biomass in the global carbon pool^19^,^24^ agroforestry may already significantly contribute to global carbon budgets^11^,^25^,^26^,^27^.

Following the guidelines of the Intergovernmental Panel on Climate Change (IPCC) for National Greenhouse Gas Inventories, Ruesch & Gibbs^28^ identified a relatively low value (5 tC ha^−1^) for agricultural land, which has been applied uniformly for Tier 1 estimates within the “Global Biomass Carbon Map for the Year 2000” dataset^28^. Although updated guidelines^29^ and more recent research^30^ provide guidance for woody biomass on croplands and quantifying carbon stocks in agricultural landscapes, the pervasive presence of trees in the agricultural landscape is still largely ignored. Here we (a) assess the significance of trees in agroforestry systems for carbon sequestration at a global level, and (b) the extent to which this resource is stable, increasing or decreasing. We test this by combining IPCC Tier 1 default estimates for carbon stored in a variety of land cover types across different bioclimatic and ecofloristic zones^28^ with previously published tree cover data based on 250 m resolution MODIS satellite remote sensing imagery^31^.

## Results

### Biomass carbon on agricultural land globally

Overall the amount of area classed as agricultural is ~22.2 million km^2^ (GLC2000)^32^. Using the IPCC Tier 1 default value, the world stores an estimated 11.1 PgC in above- and below-ground biomass carbon on agricultural land. However, in 2000 >40% of this area had ≥10% tree cover, corresponding to the FAO definition of forest. Combining the IPCC Tier 1 values with estimates of carbon storage in the hitherto ignored tree component, we produce a revised estimate of 45.3 PgC (Table 1), with trees contributing >75% (34.2 PgC) to this global total. Between 2000 and 2010 there was an additional increase of 2% tree cover, resulting in an increase of >2 PgC (or 4.6%) biomass carbon. (Our delineation of agricultural land in 2000 and 2010 remained constant (GLC 2000) to exclude any confounding trends such as agricultural expansion, abandonment, and/or forest clearing.) This gives a mean value of 20.4 tC ha^−1^ in 2000, and 21.4 tC ha^−1^ in 2010, which is more than four times larger than the IPCC Tier 1 global estimate of 5 tC ha^−1^.

There is significant variation in biomass carbon on agricultural land across regions and bioclimatic zones. The majority of agricultural areas have fairly low to moderate levels of biomass carbon: 79% (17.5 million km^2^) had <25 tC ha^−1^, and 53% (5.7 million km^2^) had <10 tC ha^−1^ (Table 2; Figs S1–S3;). By 2010 there was a further decrease of biomass carbon in areas with <10 tC ha^−1^, whilst areas with 15–40 tC ha^−1^ showed a small increase. Overall, the amount of land globally with <10 tC ha^−^1 increased by 1.3% (almost 300,000 km^2^). This resulted from a decrease of biomass carbon in areas that previously had 11–25 tC ha^−1^ (these areas decreased by 2.3%, or 520,000 km^2^). Thus, areas with low or moderate amounts of carbon experienced further decreases between 2000 and 2010, while areas with ≥26 tC ha^−1^ increased by 200,000 km^2^.

### Regional patterns

**Figure 1:**
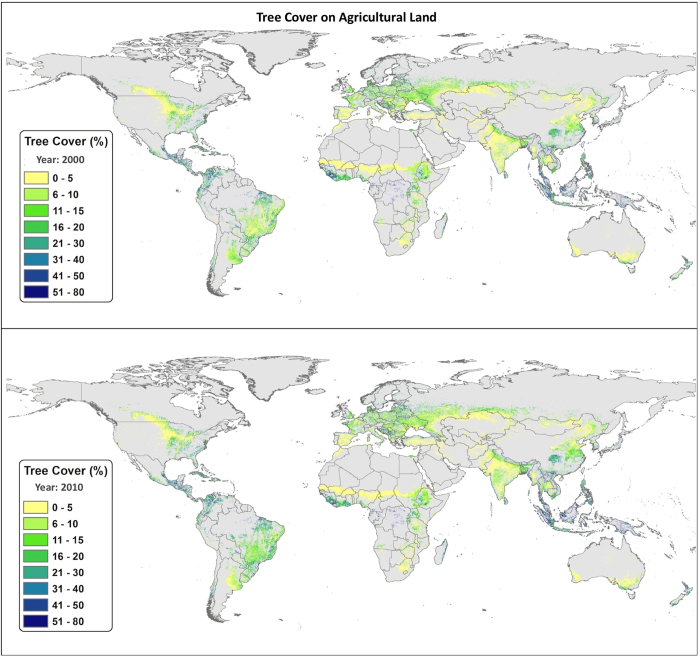
Global tree cover on agricultural land in the years 2000 and 2010. Approximately 40% of all agricultural land in the year 2010 had at least 10% tree cover (which corresponds to the FAO definition of forest). This increased by 3.7% by the 2010, to account for more than 43% of all agricultural land under some variation of agroforestry approaches. Based on this current analysis, these land-use types represent over 1 billion hectares of land and provide subsistence to more than 900 million people. Maps were produced based upon a geospatial analysis using ESRI ArcGIS software (version 10.3; http://www.esri.com/software/arcgis/arcgis-for-desktop).

**Figure 2:**
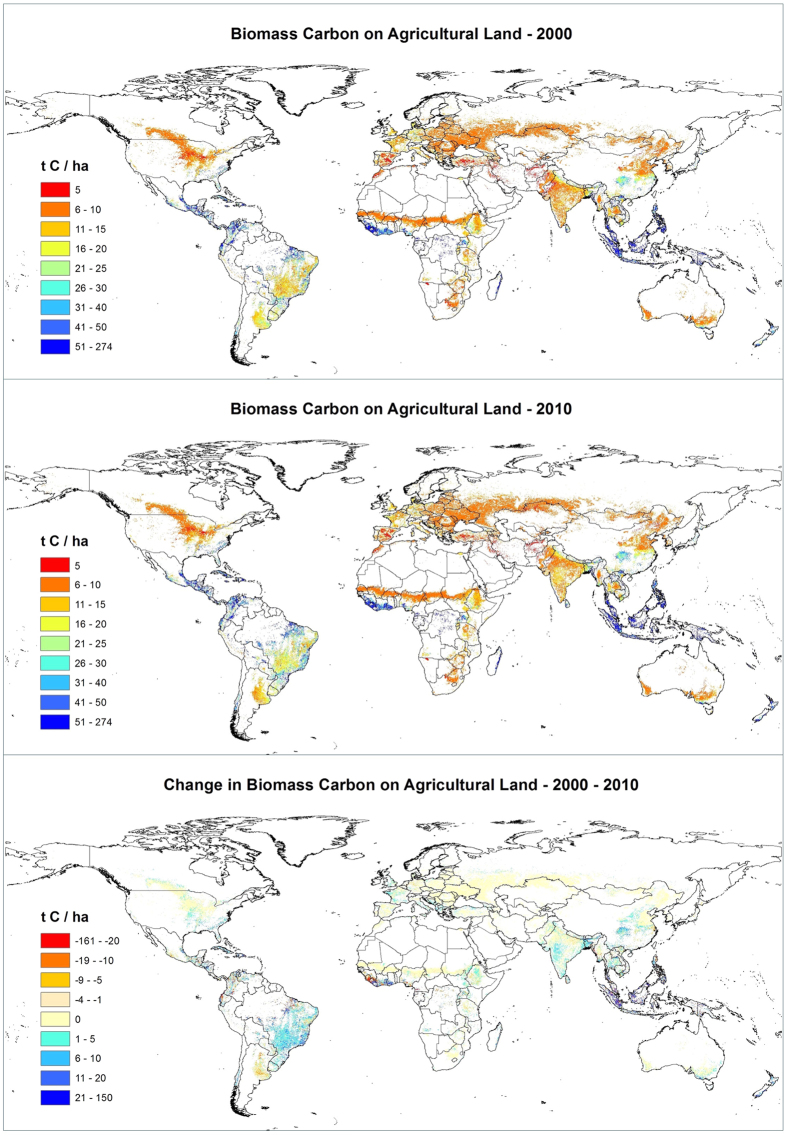
Global map of average biomass carbon per hectare on agricultural land in 2000 and 2010, and the change in average biomass carbon from 2000 to 2010 (tC ha^−1^). Maps were produced based upon a geospatial analysis using ESRI ArcGIS software (version 10.3; http://www.esri.com/software/arcgis/arcgis-for-desktop).

There are distinctive global and regional patterns (Figs S5–11) in the distribution of tree cover and biomass carbon on agricultural land in 2000 and 2010. Given the importance of the tree component for total biomass carbon we first explore the distribution of tree cover on agricultural land (Fig. 1), and then the distribution of total above-ground biomass carbon (Fig. 2).

The distribution of tree cover on agricultural land broadly followed bioclimatic zones – in particular aridity. High tree cover (>45%) was found in the humid regions such as Southeast Asia, Central America, eastern South America and central and coastal West Africa. Tree cover was moderate (10–30%) in the majority of agricultural areas in South Asia, sub-humid Africa, Central and Western Europe, Amazonian South America, and Midwestern North America. At the other extreme are agricultural areas with low (<10%) tree cover such as Eastern China, Northwest India and the Punjab, Western Asia, the southern border of the Sahara, the northern prairies of North America and Southwest Australia. Strong correlations between population density and tree cover have been shown^22^,^23^,^33^, with divergences of tree cover from climate influence stronger where population density or human activities are higher (e.g. China or India).

South America and Southeast Asia ranked highest in total above-ground biomass carbon on agricultural land with a total of 10 PgC on each continent, and they also had the greatest increase of biomass carbon (in total 1.45 PgC; ~7%). This reflects vast amounts of agricultural area, favorable climatic conditions and, in some cases, the prevalence of subsistence farming, which particularly in the tropics frequently incorporates trees. Southeast Asia had the highest biomass carbon per ha (>60 tC ha^−1^ in 2000 and 65 tC ha^−1^ in 2010). In total, only 14% of land had <10 tC ha^−1^, while >1/3 had in excess of 75 tC ha^−1^, and 26% in excess of 100 tC ha^−1^. However, there were pockets of severe decreases in Vietnam, Laos, Myanmar, and Northeast Thailand (Figs S8 and S9). Central America ranked second in terms of biomass carbon per ha with 53 tC ha^−1^ in 2000 and 56 tC ha^−1^ in 2010, and 85% of agricultural land storing >50 tC ha^−1^. South America has the greatest amount of agricultural land, but average carbon biomass was lower than in Southeast Asia (~29 tC ha^−1^ in 2000, increasing to ~31 tC ha^−1^ by 2010). However, as in Southeast Asia, only a small proportion of land had <10 tC ha^−1^ (8% in 2010 - a decrease of over 3% since 2000), while most agricultural land (51% in 2010; ~2 million km^2^) had 11–25 tC ha^−1^ (Table 2; Figs S1–3).

West and Central Africa had considerable biomass carbon (~23 tC ha^−1^), but experienced the largest proportional decline (~0.5%). South Asia and Australia/Pacific (primarily Australia) both exhibited significant increases (~8%) but their overall levels of biomass carbon on agricultural land were low with e.g. 59% (>1 million km^2^) of agricultural land in South Asia having <10 tC ha^−1^. Central Asia, North Africa, and Western Asia, along with Russia, all had <10 tC ha^−1^, reflecting arid conditions and low biomass generally. In these regions changes in biomass carbon were almost negligible.

In summary, both tree cover and consequently biomass carbon on agricultural land tend to be higher in humid regions. However, there is wide disparity between regions (Fig. S4): dry areas in both Central and South America have more biomass carbon than the global average for this level of aridity. Similarly, South and North America, West and Central Africa, Southeast Asia, and Australia/Pacific all rank above the global average for semi-humid to arid regions, while Central Asia, Russia, Europe, and South, East and West Asia have lower than expected biomass carbon. Thus, in many regions there is still potential for increasing biomass carbon on agricultural land.

### Biomass carbon on agricultural land by countries

**Figure 3:**
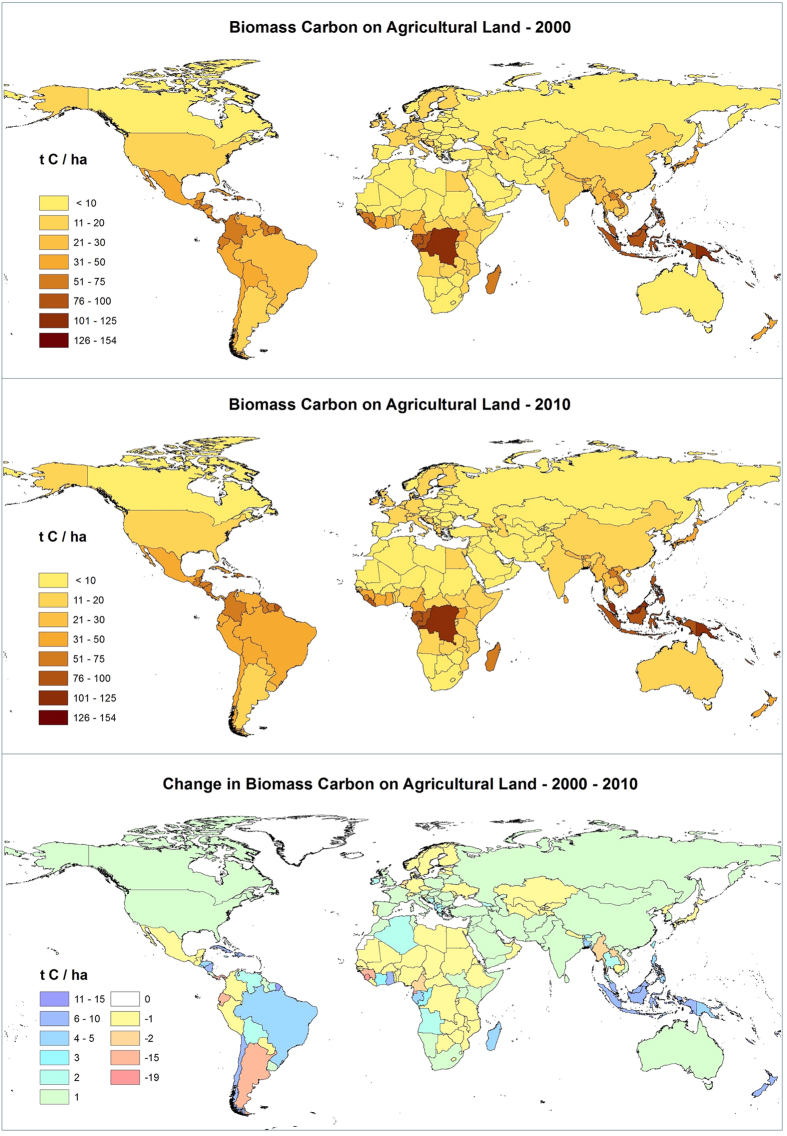
Global map of biomass carbon per hectare on agricultural land, by national average, in 2000 and 2010, and the change in national average biomass carbon on agricultural land from 2000 to 2010 (tC ha^−1^). Maps were produced based upon a geospatial analysis using ESRI ArcGIS software (version 10.3; http://www.esri.com/software/arcgis/arcgis-for-desktop).

Marked differences in biomass carbon stocks and trends over time are found among countries (Fig. 3). Twenty countries have over 0.5 PgC in biomass carbon stored on agricultural land each (Table 3). Brazil, with the greatest total amount, had nearly 6.8 PgC in 2000, which by 2010 had increased by almost 14% to 7.7 PgC. Indonesia had similarly high amounts of biomass carbon (5.5 PgC) which by 2010 had increased by more than 9%. Moderately large stocks were found in China (2.1 PgC) and India (1.73 PgC), with each increasing biomass carbon by over 7% over the decade. The top ten countries in carbon biomass storage together comprised nearly 25 PgC, although that of Colombia and D.R. Congo had slightly declined by 2010. Chile, New Zealand, Ghana, and Bangladesh’s stocks all showed increases near or in excess of 20%. In contrast, the biomass carbon stored in 23 countries declined by more than 1%, notably Sierra Leone (25%), Argentina (20%), Guinea (14%), and Myanmar (10%). In stark contrast to its neighbor Brazil (Fig. S5), Argentina’s stocks showed by far the largest decline (0.18 PgC, representing a decrease of 20%). On a per hectare basis, the corresponding change from 17.8 to 14.2 tC ha^−1^ represents a 3.6% decrease over nearly a half million km^2^ of agricultural land. Results for all countries are given in Table S1.

The largest decreases in per hectare biomass carbon were found in countries in West and Central Africa, with a more than 16 tC ha^−1^ decrease found in both Sierra Leone and Equatorial Guinea, and over 7 tC ha^−1^ decrease in Guinea, indicating a hot spot for concern (Fig. S6). Other countries with high levels of decrease (ranging from 2.0 to over 4.6 tC ha^−1^) included Egypt, the Netherlands, Cameroon, Laos, Myanmar, Panama, Argentina, and Ecuador. In Europe (Fig. S7), Switzerland, Portugal, Belgium and Germany all showed decreases of more than 1%. In total, 19 countries showed decreased per hectare biomass carbon in excess of 1 tC ha^−1^. In contrast, 41 countries showed increases greater than 1 tC ha^−1^. Some smaller countries, e.g. French Guyana, Fiji, and Vanuatu, showed increases in excess of 12 tC ha^−1^, with another 9 countries showing increases of more than 5 tC ha^−1^, notably Malaysia, Dominican Republic, New Zealand, Haiti, Indonesia, and Ghana. In all, over the decade, biomass carbon stored on agricultural land amongst the countries declined by a combined 0.63 PgC in a total of 61 countries, but increased by a combined 2.7 PgC in a total of 85 countries. (See Figs S5–11).

High average biomass carbon levels (greater than 50 tC ha^−1^ in 2010) were found in 26 countries, with particularly high levels found in some humid tropical countries, notably D.R. Congo, Papua New Guinea, Malaysia, and Indonesia. Twenty-five countries have moderate to high per hectare biomass levels between 25 and 50 tC ha^−1^, with 48 countries having low to moderate levels between 10 and 25 tC ha^−1^. In contrast, 60 countries have biomass carbon levels <10 tC ha^−1^, with the lowest levels mostly found in arid zone countries: for example, Saudi Arabia, Libya, Turkmenistan, Iraq, Afghanistan, Namibia, Kazakhstan, Jordan, Palestinian Territories, Mauritania, Moldova, Niger, Tajikistan, and Yemen all have less than 6 tC ha^−1^. However, Ukraine, Cyprus, Hungary, Mongolia, North Korea, Romania, Syria, Burkina Faso, Lesotho, and Russia also all have less than 6.5 tC ha^−1^.

## Discussion

These results show that existing tree cover - thus far ignored in most global and regional calculations - makes a major contribution to the carbon pool on agricultural lands. Consequently, a substantial and significant correction on current estimates based upon IPCC default values is warranted. If tree cover is accounted for, the total carbon estimate for agricultural land is over four times higher than when estimated with IPCC default values alone. Our estimate of >45 PcC on global cropland is within the same order of magnitude, but higher, than the 35 PgC (±9PgC) recently estimated using passive microwave remote sensing^34^. It is important to note several limitations and sources of uncertainty^35^, associated with both the remote sensing-based tree cover analysis^31^,^36^, as analysed for agricultural land by Zomer *et al*.^22^,^23^, and the conversion to and estimation of biomass carbon using the IPCC Tier 1 default values. At pixel scale (1 km^2^) the uncertainty is almost certainly unacceptably high^35^, but we are confident that the general regional and national trends established here meet the standards for the IPCC Tier 1 protocol.

Albeit small by comparison to the amount of carbon in soils (~2500 GtC)^37^, biomass carbon on agricultural land deserves attention both for its mitigation potential and its adaptation benefits. In addition, trees on agricultural land have direct impacts on the livelihoods of hundreds of millions of small farmers around the globe. Given the large amount of land potentially suitable for higher tree cover densities, sequestering carbon via increases in the tree component on agricultural land is an achievable and relatively fast route to increasing CO_2_ sequestration. Between 2000 and 2010, there was an increase of 0.2 PgC yr^−1^ of biomass carbon on agricultural land. By comparison, above-ground losses due to tropical land use conversion are currently estimated at 0.6–1.2 PgC yr^−1^ (Houghton *et al*.^18^). A strategy of enhancing climate-smart agriculture along with reducing emissions from deforestation and forest degradation via appropriate policy mechanisms thus harbours significant potential to minimise land use related carbon emissions.

In this context it is interesting to note that many places where forests are regarded as a nationally important concern, for example Indonesia or Brazil, showed both high and increasing carbon biomass on agricultural land. There is a striking distinction between Brazil and neighbouring Argentina, which experienced significant losses over the decade. In the case of Argentina, the rapid and significant decrease is likely associated with wide-spread adoption of large-scale mechanized soy production since 2001^38^,^39^. In Brazil, some of this increase may be associated with policy incentives, the abandonment or use of fallow periods on degraded pasture, and adoption of agroforestry approaches^40^,^41^,^42^. Further research is warranted to identify the drivers underlying these regional, national, and sub-national patterns, and to develop efficient policies and market mechanisms that promote both reduced forest conversion as well as adoption of enhanced carbon sequestration on agricultural lands.

As a range of recent studies have shown^27^,^27^,^43^, the benefits of increasing tree cover on agricultural land go far beyond carbon sequestration and tree-related income. Although interactions between climate and soil and their influence on crop production are complex, it is generally recognized that changes in the moisture regime (e.g. drought or heavy precipitation events) significantly influence crop productivity^44^. Soil conditions such as moisture content, temperature and nutrient levels have dramatic effects on the abundance and efficiency of N-fixing bacteria^45^, which are vitally important in cropping systems that lack fertilizer inputs^46^. These climatic conditions are mitigated by tree cover and can have a significant impact on soil fertility, which is itself a major controlling factor influencing agricultural productivity and both regional and household food security^6^. Trees are relatively permanent, and their biomass contributes to building up SOC over the longer term^10^,^11^, or improving nitrogen status through the presence of nitrogen-fixing trees^45^,^47^. Since many interactions between SOC and fertility status exhibit temperature and moisture dependent sensitivity, microclimatic modification by tree cover may also have ecosystem stabilizing and fertility benefits under changing climatic conditions^21^,^27^,^45^,^47^.

In view of the generally large potential for increasing SOC on agricultural lands, facilitation of increased SOC by tree cover, as found within many agroforestry systems, should be highlighted as an important corollary benefit^21^. The integration of a tree component appropriately on agricultural land may enhance climate resilience and/or provide multiple adaptation benefits. At the same time, there may be significant trade-offs associated with high tree cover within various specific land use types, farming systems, or with changing climatic conditions e.g. concerning productivity, food security or hydrologic balances^6^,^8^. Hundreds of millions of small farmers depend for their subsistence upon these lands, so the mitigation benefits of enhanced biomass carbon must be recognized as a significant component of an array of multiple benefits. Additional potential benefits include increased habitat and landscape connectivity for biodiversity, decreased albedo, watershed conservation and in some cases positive impacts on hydrological cycles, as well as local livelihoods. In summary, our analyses highlight that agroforestry, and tree cover on agricultural land in general, has clear potential to contribute to climate change mitigation while providing an array of adaptation benefits. In order for this potential to be unlocked and better supported, there is a need to recognize and incorporate this generally neglected carbon pool into current global and national carbon monitoring protocols.

## Methods

In order to spatially quantify georeferenced estimates of biomass carbon on agricultural land and to produce a global map of biomass carbon, a method was derived to combine (a.) a remote sensing based analysis of tree cover on agricultural land, with (b.) IPCC Tier 1 default estimates for above- and below-ground carbon stocks^28^,^48^,^49^ articulated for a variety of land cover types across a range of bioclimatic and ecofloristic zones. Results provide a global Tier-1 spatial mapping and tabulation, globally, and by global region and countries, of biomass carbon on agricultural land for the period 2000 to 2010.

The primary geodatasets used in this global analysis of biomass carbon on agricultural land are listed below:

- MOD44B MODIS Vegetation Continuous Field - Collection 5 (2000–2010): Percent Tree Cover^31^
- Global Land Cover 2000 (GLC 2000) Database^32^
- “New IPCC Tier-1 Global Biomass Carbon Map For the Year 2000”^28^
- GADM database of global administrative areas, version 2.0[J]. 2012^48^
- Aridity-Wetness Index^50^

### Assessment of global tree cover on agricultural land

In order to assess tree cover on agricultural land, a previous global analysis of tree cover which used a MODIS 250 m resolution satellite remote sensing dataset from 2000 to 2010^31^ was combined with the Global Land Cover 2000 (GLC 2000) database^32^ to extract only agricultural classes. Tree cover on agricultural land was quantified by geospatial analytical techniques, and results mapped and tabulated; globally, by region, and by countries. Detailed results of this analysis are available online in a working paper report^23^, and the geospatial dataset of tree cover on agricultural land is also available online at: http://www.worldagroforestry.org/global-tree-cover/index.html.

The MOD44B MODIS/Terra Vegetation Continuous Fields (VCF) Dataset^36^ (VCF) was developed by the University of Maryland and provides global estimates of vegetation cover in terms of woody vegetation, herbaceous vegetation and bare-ground percentages. The updated MOD44B MODIS VCF – Collection 5 dataset^31^ used in the current analysis improves upon the earlier versions and provides data at the resolution of 250 m. Three agricultural land use types from the Global Land Cover 2000 database were included in our “Agricultural Land” class:

- Cultivated and Managed Areas (agriculture — intensive),
- Cropland/Other Natural Vegetation (non-trees: mosaic agriculture/degraded vegetation).
- Cropland/Tree Cover Mosaic (agriculture/degraded forest).

The mix of tree cover over agricultural land is depicted along a continuous gradient by the MODIS VCF tree-cover dataset. Tree cover values show the percentage of the 1 km^2^ grid cell occupied (or covered) by trees, therefore, at this resolution of 1000 m^2^, the tree-cover percentage can be expressed as hectares (ha) of tree cover per km^2^. At 100% tree cover, the whole grid cell is occupied, that is, 100 ha/km^2^. To facilitate the global analysis, the VCF Tree Cover – Collection 5 dataset (250 m resolution) grid cells were aggregated to 1 km^2^ resolution. All the geodatasets were masked to exclude areas which are either non-agricultural land use types or urban areas. Tree-canopy cover on agricultural land was tabulated for all years available in the VCF- C5 dataset, that is, from 2000 to 2010. Variation in the estimates from year to year were high and not consistent with the expected year-to-year change, as could be expected from the significant variability associated with the quality of the remote-sensing dataset and seasonal and other confounding factors affecting the automatic classification algorithm used in the VCF-C5 processing. In order to reduce the effect of this variability in estimates of change during the period, we averaged the first three years of the dataset (2000–2002) and the last three years (2008–2010) and use these averaged results as the beginning and end points for the change analysis. They are further referred to within the text as 2000 and 2010, respectively, to simplify presentation of results.

### Global Tier 1 biomass carbon estimates

For Tier 1 global estimates of biomass carbon we used the “New IPCC Tier-1 Global Biomass Carbon Map For the Year 2000”^28^, available from the Carbon Dioxide Information Analysis Center (CDIAC) Oakridge National Laboratory. This global map of biomass carbon stored in above and belowground living vegetation was created using the IPCC Good Practice Guidance^48^,^49^ for reporting national greenhouse gas inventories. The global map is stratified into 124 strata (carbon zones), based on FAO ecofloristic zones, and which continent that zone is found. In each of those “carbon zones”, a carbon value has been calculated for each GLC_2000 land use class in that zone. These values are available in tables, and apply across the whole of each carbon zone.

To construct the Global Biomass Carbon Map, Ruesch and Gibbs^28^ used the IPCC GPG Tier-1 method for estimating vegetation carbon stocks using the globally consistent default values provided for aboveground biomass^49^. Belowground biomass (root) carbon stocks were added using the IPCC root to shoot ratios for each vegetation type, and total living vegetation biomass was converted to carbon stocks using the carbon fraction for each vegetation type (which varies between forests, shrublands and grasslands). All estimates and conversions were specific to each continent, ecofloristic region and vegetation type (stratified by age of forest). Thus, a total of 124 carbon zones or regions, each with a unique carbon stock value for each of the GLC_2000 Landcover Classes found in that zone were delineated, based on the IPCC Tier-1 methods and default values.

### Deriving the global Tier 1 estimates of biomass carbon on agricultural land

The IPCC Good Practice Guidance^48^ and Greenhouse Gas Inventory Guidelines^49^ provide recommendations on methods and default values for assessing carbon stocks and emissions at three tiers of detail. Following the guidelines of the Intergovernmental Panel on Climate Change (IPCC) for National Greenhouse Gas Inventories^28^, Ruesch & Gibbs^28^ identified a relatively low value (5 tC ha^−1^) for agricultural land, which has been applied uniformly and globally for Tier 1 estimates within the “Global Biomass Carbon Map for the Year 2000” dataset.

In order to account for the added contribution of tree cover on agricultural land, we used the default Tier 1 biomass carbon value for agricultural land (5tC/ha) as the baseline value, i.e. at 0% tree cover the biomass carbon is 5tC/ha (in all carbon zones). We then used the biomass carbon value of the GLC_2000 Mixed Forest class (or similar class in case this class is not present) in that same carbon zone as a surrogate biomass carbon value where there is full tree cover on agricultural land (i.e. tree cover percentage = 100). We then assume a linear increase in biomass carbon from 0 to 100 percent tree cover where, within a specific grid cell in a specific carbon zone:

- Biomass carbon is equal to the default Tier 1 value for agricultural land (5 tC/ha) when there are no trees on that land, ◦ (i.e. tree cover = 0%).
- There is an incremental linear increase of tC/ha proportionally as tree cover increases from the baseline (5 tC/ha at 0% tree cover) up to the maximum value for Mixed Forest in that specific carbon zone, ◦ (i.e. biomass carbon values on agricultural land with 100% tree cover are equal to the related Mixed Forest class).

Results were tabulated and mapped globally, by global region, and by country. All results datasets from this analysis of carbon biomass on agricultural land are available online at http://www.worldagroforestry.org/global-tree-cover/index.html. An expanded Methods Section is provided in the Supplementary Information.

## Additional Information

**How to cite this article**: Zomer, R. J. *et al*. Global Tree Cover and Biomass Carbon on Agricultural Land: The contribution of agroforestry to global and national carbon budgets. *Sci. Rep.* **6**, 29987; doi: 10.1038/srep29987 (2016).

## Supplementary Material

Supplementary Information

## Figures and Tables

**Table 1:** Total biomass carbon on agricultural land (in PgC; and as a percentage of the total biomass carbon in 2000) and average per hectare biomass carbon (tC/ha) in the year 2000 and 2010 globally and by region, and the contribution by trees to biomass carbon on agricultural land.

Biomass Carbon on Agricultural Land								
Region	Total Biomass Carbon				Average Biomass Carbon			Total Agricultural Area (km^2^)
	Pg C			Increase as % of Total C	t C/ha			
	2000	2010	Change		2000	2010	Change	
Australia/Pacific	2.11	2.28	0.17	*8.06*	26.7	28.9	*2.2*	790,658
Central America	1.42	1.52	0.09	*6.45*	52.9	56.3	*3.4*	269,235
Central Asia	0.48	0.47	0.00	*−1.04*	5.7	5.7	*−0.1*	830,949
East Asia	2.37	2.53	0.16	*6.95*	13.2	14.1	*0.9*	1,795,893
Eastern and Southern Africa	2.31	2.30	0.00	*−0.17*	14.7	14.6	*−0.0*	1,573,527
Europe	2.13	2.15	0.02	*0.96*	9.3	9.4	*0.1*	2,299,766
North Africa	0.11	0.11	0.00	*−0.01*	7.3	7.3	*−0.0*	155,948
North America	3.31	3.40	0.09	*2.68*	16.0	16.4	*0.4*	2,073,033
Russia	1.07	1.07	0.00	*0.02*	6.4	6.4	*0.0*	1,669,166
South America	11.34	12.13	0.79	*6.95*	29.2	31.2	*2.0*	3,888,792
South Asia	2.30	2.48	0.18	*7.85*	12.6	13.6	*1.0*	1,827,025
South East Asia	10.03	10.69	0.66	*6.59*	60.8	64.8	*4.0*	1,648,268
West and Central Africa	5.57	5.45	−0.12	*−2.18*	23.3	22.8	*−0.5*	2,390,980
Western Asia	0.75	0.79	0.04	*4.72*	7.9	8.2	*0.4*	955,689
**Global**	**45.30**	**47.37**	**2.07**	***4.57***	**28.0**	**29.0**	***0.95***	**22,168,929**
*Agricultural Baseline*	*11.08*	*11.08*			*5.0*	*5.0*		
**Contribution by Trees**	**34.22**	**36.29**	**2.07**	***4.57***	**23.03**	**23.97**	***0.95***	

There has been a substantial increase (>2 PgC) in total biomass carbon being stored on agricultural land globally, with a corresponding increase in average biomass carbon hectare (from 20.4 to 21.4 tC ha^−1^). More than 75% of that was contributed by the tree component. South America and Southeast Asia have by far the largest carbon stocks on agricultural land.

**Table 2:** Areal extent of agricultural land (km^2^) as found within a set of biomass carbon classes (tC ha^−1^) in the year 2000 and 2010, by region, and the change in areal extent of agricultural land within these classes between the years 2000 and 2010.

Areal Extent of Agricultural Land within Biomass Carbon Class - 2000												
Biomass C (t/ha)	<=10		11–25		26–50		51–75		76–100		>100	
	km^2^	***%***	km^2^	***%***	km^2^	***%***	km^2^	***%***	km^2^	***%***	km^2^	***%***
Region												
Australia/Pacific	1,278,323	*62*	448,373	*22*	204,637	*10*	94,318	*5*	28,082	*1*	19,300	*1*
Central America	2,127	*1*	42,684	*16*	109,089	*41*	60,978	*23*	27,172	*10*	27,185	*10*
Central Asia	815,839	*98*	14,780	*2*	330	*0*	—	—	—	—	—	—
East Asia	1,081,961	*60*	444,831	*25*	243,055	*14*	25,484	*1*	562	*0*	—	*-*
Eastern and Southern Africa	765,194	*49*	635,878	*40*	114,282	*7*	33,601	*2*	16,242	*1*	8,330	*1*
Europe	1,685,506	*73*	532,058	*23*	80,826	*4*	1,376	*0*	—	—	—	—
North Africa	468,621	*59*	119,134	*15*	82,632	*10*	38,960	*5*	18,302	*2*	63,009	*8*
North America	135,591	*87*	20,029	*13*	328	*0*	—	—	—	—	—	—
Russia	1,589,333	*95*	76,478	*5*	3,336	*0*	19	*0*	—	—	—	—
South America	438,511	*11*	2,010,707	*52*	858,396	*22*	323,129	*8*	124,265	*3*	133,784	*3*
South Asia	1,077,780	*59*	620,558	*34*	83,891	*5*	21,555	*1*	12,733	*1*	10,508	*1*
SouthEast Asia	225,389	*14*	284,365	*17*	291,315	*18*	257,278	*16*	211,736	*13*	378,185	*23*
West and Central Africa	1,469,661	*61*	344,741	*14*	233,506	*10*	126,778	*5*	79,968	*3*	136,326	*6*
Western Asia	820,847	*86*	101,556	*11*	28,923	*3*	4,353	*0*	10	*0*	—	—
Global Total	11,854,683	*53*	5,696,172	*26*	2,334,546	*11*	987,829	*4*	519,072	*2*	776,627	*4*
Areal Extent of Agricultural Land within Biomass Carbon Class - 2010												
Australia/Pacific	476,741	*60*	102,461	*13*	73,334	*9*	48,337	*6*	22,538	*3*	67,247	*9*
Central America	1,621	*1*	34,942	*13*	106,168	*39*	68,229	*25*	30,360	*11*	27,915	*10*
Central Asia	816,972	*98*	13,678	*2*	299	*0*	—	—	—	—	—	—
East Asia	1,066,727	*59*	438,840	*24*	255,198	*14*	34,410	*2*	718	*0*	—	—
Eastern and Southern Africa	881,523	*56*	534,088	*34*	96,050	*6*	37,796	*2*	16,558	*1*	7,512	*0*
Europe	1,809,606	*79*	406,180	*18*	83,223	*4*	757	*0*	—	—	—	—
North Africa	135,905	*87*	19,941	*13*	102	*0*	—	—	—	—	—	—
North America	1,313,470	*63*	411,039	*20*	204,567	*10*	96,319	*5*	29,221	*1*	18,417	*1*
Russia	1,596,327	*96*	69,913	*4*	2,915	*0*	11	*0*	—	—	—	—
South America	321,760	*8*	1,964,145	*51*	1,028,356	*26*	334,189	*9*	124,305	*3*	116,037	*3*
South Asia	1,086,309	*59*	588,574	*32*	109,596	*6*	22,977	*1*	10,740	*1*	8,829	*0*
SouthEast Asia	226,738	*14*	266,556	*16*	292,212	*18*	249,594	*15*	186,845	*11*	426,323	*26*
West and Central Africa	1,573,021	*66*	255,654	*11*	217,659	*9*	151,654	*6*	72,708	*3*	120,284	*5*
Western Asia	842,890	*88*	76,679	*8*	31,139	*3*	4,948	*1*	33	*0*	—	—
Global Total	12,149,610	*55*	5,182,690	*23*	2,500,818	*11*	1,049,221	*5*	494,026	*2*	792,564	*4*
Change in Areal Extent of Agricultural Land within Biomass Carbon Class - 2000 to 2010												
Australia/Pacific	−801,582	−*1.4*	−345,912	−*8.7*	−131,303	−*0.6*	−45,981	*1.6*	−5,544	*1.5*	47,947	*7.6*
Central America	−506	−*0.2*	−7,742	−*2.9*	−2,921	−*1.1*	7,251	*2.7*	3,188	*1.2*	730	*0.3*
Central Asia	1,133	*0.1*	−1,102	−*0.1*	−31	−*0.0*	0	*0.0*	0	*0.0*	0	*0.0*
East Asia	−15,234	−*0.8*	−5,991	−*0.3*	12,143	*0.7*	8,926	*0.5*	156	*0.0*	0	*0.0*
Eastern and Southern Africa	116,329	*7.4*	−101,790	−*6.5*	−18,232	−*1.2*	4,195	*0.3*	316	*0.0*	−818	−*0.1*
Europe	124,100	*5.4*	−125,878	−*5.5*	2,397	*0.1*	−619	−*0.0*	0	*0.0*	0	*0.0*
North Africa	−332,716	*27.9*	−99,193	−*2.3*	−82,530	−*10.4*	−38,960	−*4.9*	−18,302	−*2.3*	−63,009	−*8.0*
North America	1,177,879	−*23.6*	391,010	*7.0*	204,239	*9.7*	96,319	*4.6*	29,221	*1.4*	18,417	*0.9*
Russia	6,994	*0.4*	−6,565	−*0.4*	−421	−*0.0*	−8	−*0.0*	0	*0.0*	0	*0.0*
South America	−116,751	−*3.0*	−46,562	−*1.2*	169,960	*4.4*	11,060	*0.3*	40	*0.0*	−17,747	−*0.5*
South Asia	8,529	*0.5*	−31,984	−*1.8*	25,705	*1.4*	1,422	*0.1*	−1,993	−*0.1*	−1,679	−*0.1*
SouthEast Asia	1,349	*0.1*	−17,809	−*1.1*	897	*0.1*	−7,684	−*0.5*	−24,891	−*1.5*	48,138	*2.9*
West and Central Africa	103,360	*4.3*	−89,087	−*3.7*	−15,847	−*0.7*	24,876	*1.0*	−7,260	−*0.3*	−16,042	−*0.7*
Western Asia	22,043	*2.3*	−24,877	−*2.6*	2,216	*0.2*	595	*0.1*	23	*0.0*	0	*0.0*
Global Total	294,927	*1.3*	−513,482	−*2.3*	166,272	*0.8*	61,392	*0.3*	−25,046	−*0.1*	15,937	*0.1*

The majority of agricultural land has <10 tC ha^−1^. Area with <10 tC ha^−1^ increased by 1.3%, area with between 11–25 tC ha^−1^ decreased by 2.3%, while area >25 tC ha^−1^ generally increased.

**Table 3:** Top countries as ranked by the greatest increase and greatest decrease in total (above and below ground) biomass carbon (tC) on agricultural land, and as a percentage of total biomass carbon in the year 2000.

Country	Total Biomass Carbon (million t C)				Average Biomass Carbon			Total Agricultural Area (km^2^)
	t C * 10^6^			Increase as % of Total	t C/ha			
	2000	2010	Change		2000	2010	Change	
Brazil	6790	7729	938.8	13.8	26.8	30.5	3.7	2,535,884
Indonesia	5493	6007	513.9	9.4	88.3	96.6	8.3	621,762
China	2153	2320	166.4	7.7	12.7	13.7	1.0	1,692,194
India	1834	1970	136.0	7.4	11.2	12.0	0.8	1,640,067
Malaysia	1034	1140	105.8	10.2	96.9	106.8	9.9	106,718
United States	1727	1804	76.8	4.4	12.6	13.2	0.6	1,366,923
New Zealand	309	381	72.1	23.3	40.2	49.6	9.4	76,785
Philippines	1394	1458	64.7	4.6	73.7	77.1	3.4	189,123
Ghana	271	332	61.1	22.6	35.6	43.7	8.1	75,900
Papua New Guinea	1094	1140	46.0	4.2	105.6	110.0	4.4	103,583
Coate d’Ivoire	641	684	43.7	6.8	42.9	45.8	2.9	149,304
Thailand	696	738	41.9	6.0	25.3	26.8	1.5	275,437
Bangladesh	207	248	41.0	19.8	20.3	24.4	4.0	101,875
Australia	595	635	39.9	6.7	10.0	10.6	0.7	597,768
Chile	153	189	35.7	23.4	31.2	38.5	7.3	49,040
Cuba	268	302	34.0	12.7	43.7	49.3	5.6	61,207
Venezuela	647	677	29.4	4.5	44.0	46.0	2.0	147,179
Madagascar	396	420	24.1	6.1	59.8	63.4	3.6	66,178
Nicaragua	191	211	20.1	10.5	48.5	53.6	5.1	39,408
Turkey	263	282	18.5	7.0	9.0	9.6	0.6	291,990
Kazakhstan	270	265	−4.6	−1.7	5.5	5.4	−0.1	492,142
Cambodia	102	96	−5.7	−5.6	18.2	17.2	−1.0	55,846
Colombia	1499	1493	−5.8	−0.4	52.7	52.4	−0.2	284,672
Guatemala	262	256	−6.3	−2.4	63.3	61.8	−1.5	41,401
Equatorial Guinea	40	33	−7.0	−17.6	93.8	77.3	−16.5	4,273
Panama	144	134	−9.6	−6.7	53.2	49.6	−3.6	26,989
Laos	248	236	−11.8	−4.8	54.0	51.4	−2.6	45,916
Chad	191	179	−12.0	−6.3	7.4	6.9	−0.5	257,824
Paraguay	235	221	−14.2	−6.0	29.1	27.4	−1.8	80,714
Tanzania	264	248	−15.9	−6.0	11.6	10.9	−0.7	226,692
Cameroon	225	209	−16.6	−7.4	34.0	31.5	−2.5	66,206
Ethiopia	584	564	−19.8	−3.4	13.4	13.0	−0.5	435,230
D. R. Congo	1372	1350	−21.8	−1.6	113.0	111.2	−1.8	121,384
Germany	280	256	−23.5	−8.4	16.5	15.1	−1.4	169,860
Nigeria	545	513	−31.6	−5.8	12.8	12.1	−0.7	425,128
Ecuador	413	376	−37.6	−9.1	50.6	46.0	−4.6	81,712
Guinea	352	303	−49.0	−13.9	52.2	45.0	−7.3	67,377
Myanmar	553	496	−57.2	−10.3	28.7	25.7	−3.0	193,138
Sierra Leone	370	279	−91.2	−24.7	67.7	51.0	−16.7	54,642
Argentina	874	699	−175.0	−20.0	17.8	14.2	−3.6	491,951

Total biomass carbon is given in millions of tons of carbon (tC * 10^6^) and average biomass carbon in tons of carbon per hectare (tC/ha). Brazil, Indonesia, China and India had the largest amounts of biomass carbon stored on agricultural land, while New Zealand, Chile, and Ghana had the highest rates of percentage increases. Argentina, Sierra Leone, Myanmar, and Guinea had the highest losses in carbon stocks.
