# Supplementary material for: Global Tree Cover and Biomass Carbon on Agricultural Land: The contribution of agroforestry to global and national carbon budgets
Source: Sci Rep. 2016 Jul 20;6:29987. doi: 10.1038/srep29987 (PMC4951720; doi:10.1038/srep29987)
Supplement: Supplementary Information [file srep29987-s1.pdf]

Global Tree Cover and Biomass Carbon on Agricultural Land:

The contribution of agroforestry to global and national carbon budgets

Supplementary Information

May 14, 2016

Submitted to:

Scientific Reports

Authors:

Robert J. Zomer[1,2], Henry Neufeldt[3], Jianchu Xu[1,2] *, Antje Ahrends[4], Deborah Bossio[5], Antonio Trabucco[6,7], Meine van Noordwijk[8,9], Mingcheng Wang[1]

1. *Key Laboratory for Plant Diversity and Biogeography of East Asia (KLPB), Kunming Institute of Botany, Chinese Academy of Science, Kunming 650201, Yunnan, China*
2. *Centre for Mountain Ecosystem Studies, World Agroforestry Center (ICRAF), East and Central Asia Region, Kunming 650201, China*
3. *World Agroforestry Centre (ICRAF), Nairobi, Kenya*
4. *Royal Botanic Garden Edinburgh, 20A Inverleith Row, Edinburgh, UK, EH3 5LR*
5. *International Center for Tropical Agriculture (CIAT), Soils Division, Nairobi, Kenya*
6. *Euro-Mediterranean Center on Climate Change, IAFES Division, Sassari, Italy. Email:*
7. *Department of Science for Nature and Environmental Resources (DIPNET), University of Sassari, Via De Nicola 9, 07100 Sassari, Italy*
8. *World Agroforestry Center (ICRAF) – Southeast Asia Regional Office, Bogor, Indonesia*
9. *Wageningen University and Research, Plant Production Systems, Wageningen, the Netherlands*

* Corresponding Author – Email: J.C.Xu@cgiar.org

Supporting Information - Methods:

To quantify estimates of biomass carbon on agricultural land, IPCC Tier 1 default estimates for carbon stored in a variety of land cover types across bioclimatic strata were combined with tree cover estimates based on 250 m resolution MODIS satellite imagery, to provide a global Tier-1 spatial mapping and tabulation, by region and countries, of biomass carbon on agricultural land for the period 2000-2010.

The spatial modeling procedure was developed and implemented in ArcGIS 10.2 (ESRI Inc.) using both ArcAML and Python programming language. Datasets were re-projected to a sinusoidal projection (World Sinusoidal) in order to calculate zonal statistics and carry out areal computations, as it represents area extent accurately across latitudes (i.e., equal-area projection). The cell size for analyses in sinusoidal projection is 1 km^2., These datasets are presented in geographic coordinates in the figures, for mapping and presentation purposes.

Assessment of Global Tree Cover on Agricultural Land:

The global geospatial analysis to identify tree cover on agricultural land combined a global assessment of tree cover, based upon a MODIS 250 m resolution satellite remote sensing dataset [31], with the Global Land Cover 2000 (GLC 2000) land-use classification . Tree cover on agricultural land was identified and results mapped and tabulated; globally, by global region, and by countries. A detailed description of this analysis is available online in a working paper report (Zomer et al, 2014):

http://www.worldagroforestry.org/sites/default/files/WP89_text_only.pdf

Geodatasets

The geodatasets used in the analysis are listed below.

- MOD44B MODIS Vegetation Continuous Field - Collection 5 (2000 through to 2010):
  - Percent Tree Cover (DiMiceli et al 2011)
- Global Land Cover 2000 (GLC 2000) Database (Bartholomé and Belward, 2005)
- GADM database of global administrative areas, version 2.0[J]. 2012. (GADM, 2012)
- Aridity-Wetness Index (Zomer et al 2007)
  -

Tree-cover data

The MOD44B MODIS/Terra Vegetation Continuous Fields Dataset (VCF) (Hansen 2003)

was developed by the University of Maryland and provides global estimates of vegetation cover in terms of woody vegetation, herbaceous vegetation and bare-ground percentages. The updated MOD44B MODIS VCF – Collection 5 dataset (DiMiceli et al 2011) used in the current analysis improves upon the earlier versions and provides data at the resolution of 250 m. A limited amount of validation performed using field data from two sites in Maryland and three sites in Brazil, South America show that the Collection 5VCF product is substantially more accurate then previous versions, with accuracy significantly improved within agricultural areas and forest clearings. (User Guide for VCF Collection 5 – Version 1). This data (and a User Guide for VCF Collection 5 – Version 1) is available online at:

http://www.landcover.org/data/vcf/

Land-cover categories

Three agricultural land-use types from the Global Land Cover Class scheme used for the Global Land Cover 2000 database were selected as relevant for the specific objectives of this work:

- Cultivated and Managed Areas (agriculture — intensive),
- Cropland/Other Natural Vegetation (non-trees: mosaic agriculture/degraded vegetation)
- Cropland/Tree Cover Mosaic (agriculture/degraded forest).

Although at first the Cropland/Tree Cover Mosaic type seems to identify agroforestry systems, the mix of forest and agriculture does not occur at discrete intervals but is a gradient where the two components of landscape-level agroforestry mix within the landscape. The mix of tree cover over agriculture land is depicted along a continuous gradient by the MODIS VCF tree-cover dataset, within the relevant GLC2000 land-cover type. Tree cover shows the percentage of the 1 km^2 grid cell occupied by trees, therefore, at this resolution of 1000 m^2, the tree-cover percentage can be expressed as hectares (ha) of tree cover per km^2. At 100% tree cover, the whole grid cell is occupied, that is, 100 ha/km^2.

The Global Land Cover 2000 database is available online here:

http://www.gvm.jrc.it/glc2000

Administrative boundaries

The GADM database of Global Administrative Areas was used to define both regional and country boundaries. The GADM database is available online here:

http://www.gadm.org

Aridity–Wetness Index

A global model of aridity (Zomer et al. 2007) was used to stratify ecological conditions based on climatic and agro-ecological characteristics. Aridity is expressed as a function of precipitation, temperature and potential evapotranspiration (PET). Based upon an attempt to classify climatic zones by moisture regime, the Aridity–Wetness Index (AWI) quantifies precipitation deficit over atmospheric water demand as:

- Aridity–Wetness Index (AWI) = MAP / MAE] where:
  - MAP = mean annual precipitation
  - MAE = mean annual evapotranspiration

The AWI dataset is available online here:

http://csi.cgiar.org/aridity/

Global Tree Cover on Agricultural Land

To facilitate the global analysis, the VCF Tree Cover – Collection 5 dataset (250 m resolution) grid cells were aggregated to 1 km^2 resolution. All the geodatasets were masked to exclude areas which are either non-agricultural land-use types or urban areas.

Tree-canopy cover on agricultural land has been tabulated for all years available in the VCF- C5 dataset, that is, from 2000 to 2010. Variation in the estimates from year to year appears to be high and not consistent with the expected year-to-year change. There is a fair amount of 'noise' in the year-to-year estimates, which can be expected from having a significant variability associated with the quality of the remote-sensing dataset and seasonal and other confounding factors affecting the classification algorithm used in the VCF-C5 processing. In order to reduce the effect of this variability in estimates of change during the period, we have averaged the first three years of the dataset (2000–2002) and the last three years (2008–2010) and use these averaged results as the beginning and end points for the change analysis. They are further referred to within the text as 2000 and 2010, respectively, to simply presentation of the results.

.

The extent of agricultural land and various associated tree-canopy cover values have been analyzed, compared, mapped and tabulated globally, and by global regions, countries, and aridity-wetness index zones. Within each stratum, or within specific aggregation of strata, zonal statistical values (mean, sum, total area, percentiles, areal distribution etc) were summarized to describe those factors of interest for this study.

Cumulative agricultural area is presented at decreasing tree-canopy cover to infer at global and subcontinent scales the total area engaged above any specific tree-canopy cover values. In a second stage, the same cumulative distribution of total agricultural

land in function of tree-canopy cover has been disaggregated for five different aridity classes (AWI < 0.45 or arid, 0.45 < AWI < 0.6 or semi-arid, 0.6 < AWI < 0.8 or sub-humid, 0.8 < AWI < 1.0 or humid, AWI >1.0 or very humid) to show how climate regimes might differentiate specific patterns of interdependence between tree-canopy cover and bioclimatic conditions for different geographical areas.

Detailed methodology and analytical results from this analysis are available online in a ICRAF Working Paper (Zomer et al. 2014) at:

http://www.worldagroforestry.org/downloads/Publications/PDFS/WP14064.pdf

Spatial datasets from this global analysis of tree cover on agricultural land are available online at:

http://www.worldagroforestry.org/global-tree-cover/index.html

Global Biomass Carbon Estimates:

For Tier 1 global estimates of biomass carbon we used the “New IPCC Tier-1 Global Biomass Carbon Map For the Year 2000” (Ruesch and Gibbs, 2008) available from the Carbon Dioxide Information Analysis Center (CDIAC) Oakridge National Laboratory. This spatial delineation and global map of biomass carbon stored in above and belowground living vegetation was created using the International Panel on Climate Change (IPCC) Good Practice Guidance (Penman et al 2003; IPCC 2006) for reporting national greenhouse gas inventories. The global map is stratified into 124 strata (carbon zones), based on FAO ecofloristic zones, and which continent that zone is found. In each of those “carbon zones” a carbon value has been calculated for each GLC_2000 landuse class in that zone. These values are available in tables, and apply across the whole of each carbon zone.

The authors state that this “… spatial database is likely the best available, globally-consistent map depicting vegetation carbon stocks, circa 2000”. It is based on widely accepted IPCC methods for estimating carbon stocks at the national level. However, the methods employed were not directly linked to ground-based measures of carbon stocks and have not been validated with field data. It is noted that croplands received the same carbon stock value regardless of the type of crop that might be growing.

To construct the Global Biomass Carbon Map, Ruesch and Gibbs (2008) used the IPCC GPG Tier-1 method for estimating vegetation carbon stocks using the globally consistent default values provided for aboveground biomass (IPCC 2006). Belowground biomass (root) carbon stocks were added using the IPCC root to shoot ratios for each vegetation type, and then total living vegetation biomass was converted to carbon stocks using the carbon fraction for each vegetation type (which varies between forests, shrublands and grasslands). All estimates and conversions were specific to each continent, ecoregion

and vegetation type (stratified by age of forest). Thus, a total of 124 carbon zones or regions, each with a unique carbon stock value for each of the GLC_2000 Landcover Classes found in that zone, were delineated, based on the IPCC Tier-1 methods and default values.

Available online from the Carbon Dioxide Information Analysis Center, Oak Ridge National Laboratory, Oak Ridge, Tennessee.

http://cdiac.ornl.gov/epubs/ndp/global_carbon/carbon_documentation.html

Deriving the Global Tier 1 Estimates of Biomass Carbon on Agricultural Land:

The IPCC Good Practice Guidance (Penman et al 2003) and Greenhouse Gas Inventory Guidelines (IPCC 2006) provide recommendations on methods and default values for assessing carbon stocks and emissions at three tiers of detail. Following the guidelines of the Intergovernmental Panel on Climate Change (IPCC) for National Greenhouse Gas Inventories [28] Ruesch & Gibbs (2008) identified a relatively low value (5 t C ha^{-1}) for agricultural land, which has been applied uniformly and globally for Tier 1 estimates within the “Global Biomass Carbon Map for the Year 2000” dataset (Ruesch & Gibbs, 2008).

In order to account for the added contribution of tree cover on agricultural land, we use the default Tier 1 biomass carbon value for agricultural land (5tC/ha) as the baseline value, i.e. at 0% treecover the biomass carbon is 5tC/ha (in all carbon zones).

We use the biomass carbon value of the GLC_2000 Mixed Forest class (or similar class in case this class is not present) in that same carbon zone as a surrogate biomass carbon value where there is full tree cover on agricultural land (i.e. tree cover percentage = 100). We then assume a linear increase in biomass carbon from 0 to 100 percent tree cover where, within a specific grid cell in a specific carbon zone:

- Biomass carbon is equal to the default tier 1 value for agricultural land (5 tC/ha) when there are no trees on that land,
  - (i.e. tree cover = 0%)
- There is an incremental linear increase of tC/ha proportionally as tree cover increases up to the maximum value for Mixed Forest in that specific carbon zone,
  - (i.e. biomass carbon values on agricultural land with 100% tree cover are equal to the related Mixed Forest class.)

Results were tabulated and mapped globally, by region, and by country. Spatial datasets resulting from this global analysis of carbon biomass on agricultural land are available online at:

http://www.worldagroforestry.org/global-tree-cover/index.html

Supporting Information – Figures and Tables:

Figure S1: Average biomass carbon per hectare (tC ha^{-1}) shown by the cumulative area of agricultural land (km^2) with that amount of average biomass carbon, and as a percent (%) of total agricultural land globally. About 79% (17.5 million km^2) had <25 t C ha^{-1}, and 53% (5.7 million km^2) had <10 t C ha^{-1}.

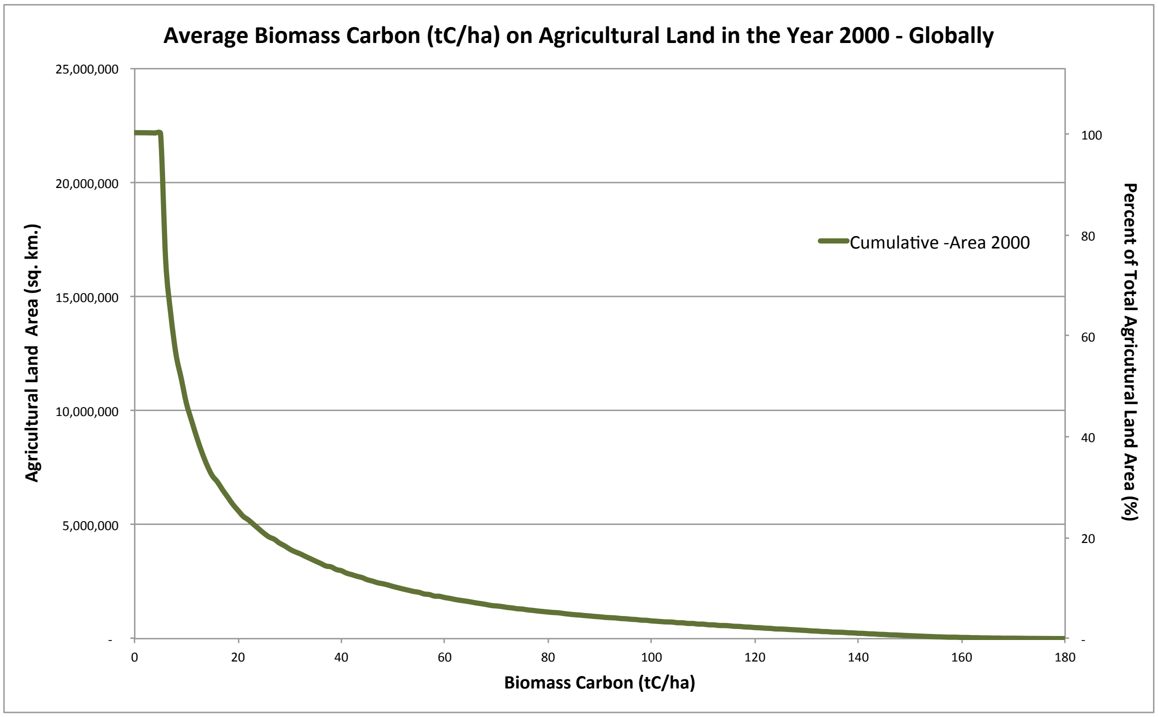

Figure S2: Areal extent of agricultural land (km2) as found within a set of biomass carbon classes (tC ha-1) in the year 2000 and 2010, by region, and the change in areal extent of agricultural land within these classes between the years 2000 and 2010. (Graphical representation of Table 2 in text).

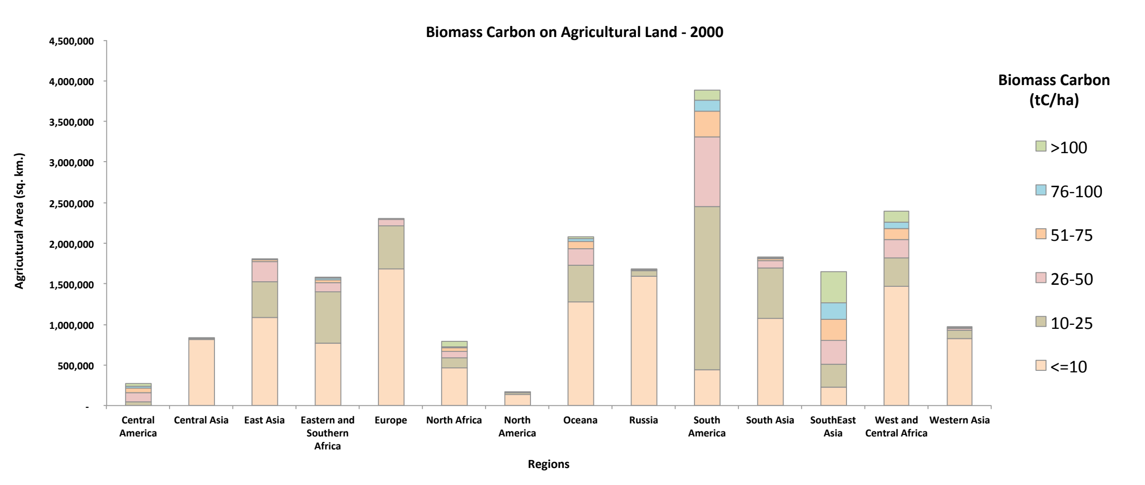

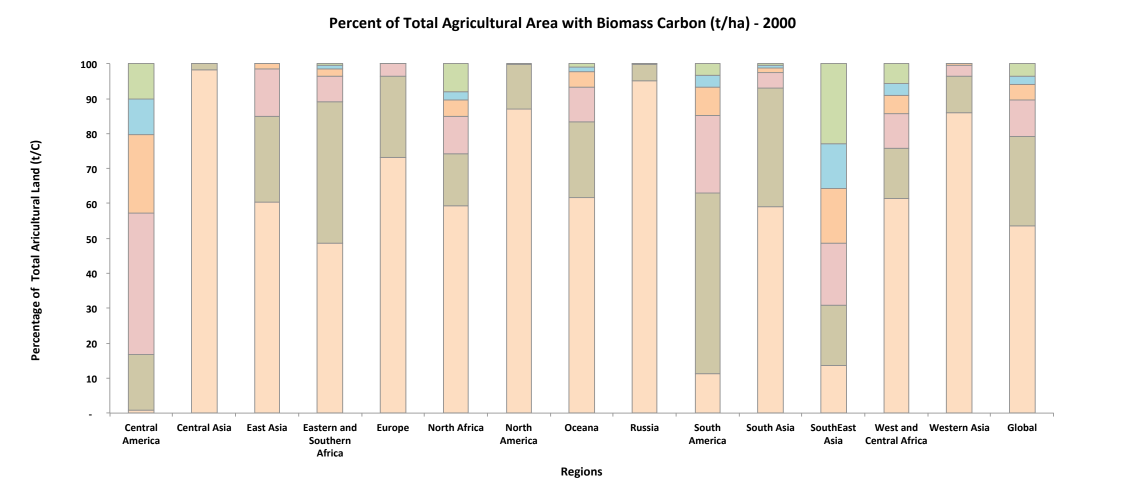

Biomass Carbon on Agricultural Land - 2000

Biomass C (t/ha)	<=10		11-25		26-50		51-75		76-100		>100	
	km^2	%	km^2	%	km^2	%	km^2	%	km^2	%	km^2	%
**Region**												
Central America	2,127	1	42,684	16	109,089	41	60,978	23	27,172	10	27,185	10
Central Asia	815,839	98	14,780	2	330	0	-	-	-	-	-	-
East Asia	1,081,961	60	444,831	25	243,055	14	25,484	1	562	0	-	-
Eastern and Southern Africa	765,194	49	635,878	40	114,282	7	33,601	2	16,242	1	8,330	1
Europe	1,685,506	73	532,058	23	80,826	4	1,376	0	-	-	-	-
North Africa	468,621	59	119,134	15	82,632	10	38,960	5	18,302	2	63,009	8
North America	135,591	87	20,029	13	328	0	-	-	-	-	-	-
Oceana	1,278,323	62	448,373	22	204,637	10	94,318	5	28,082	1	19,300	1
Russia	1,589,333	95	76,478	5	3,336	0	19	0	-	-	-	-
South America	438,511	11	2,010,707	52	858,396	22	323,129	8	124,265	3	133,784	3
South Asia	1,077,780	59	620,558	34	83,891	5	21,555	1	12,733	1	10,508	1
SouthEast Asia	225,389	14	284,365	17	291,315	18	257,278	16	211,736	13	378,185	23
West and Central Africa	1,469,661	61	344,741	14	233,506	10	126,778	5	79,968	3	136,326	6
Western Asia	820,847	86	101,556	11	28,923	3	4,353	0	10	0	-	-
**Global Total**	11,854,683	53	5,696,172	26	2,334,546	11	987,829	4	519,072	2	776,627	4

Figure S3: Areal extent of agricultural land (km2) as found within a set of biomass carbon classes (tC ha-1) in the year 2000 and 2010, by region, and the change in areal extent of agricultural land within these classes between the years 2000 and 2010. The majority of agricultural land has < 10 t C ha-1. (Graphical representation of Table 2 in text).

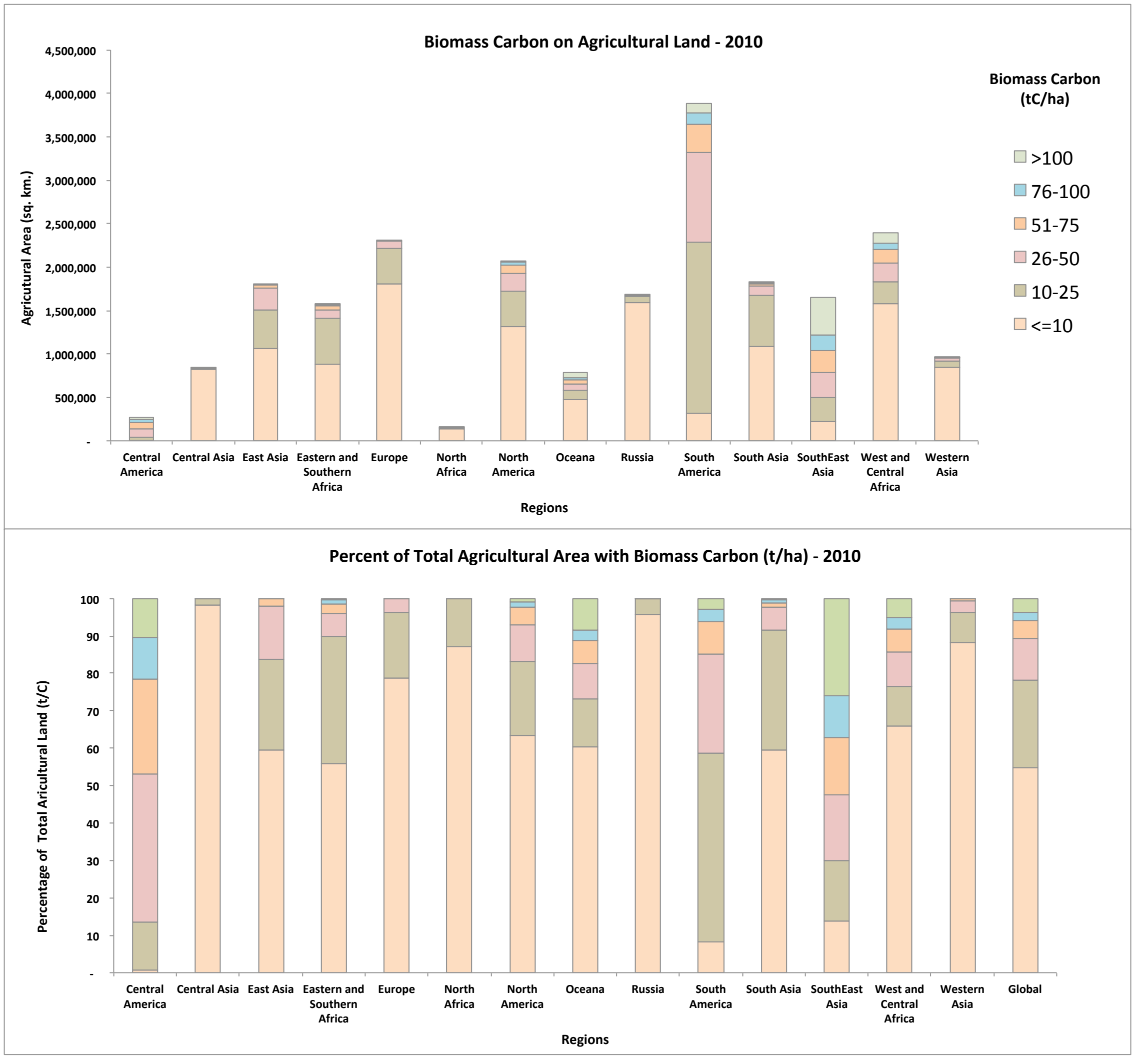

Biomass Carbon on Agricultural Land - 2010

Biomass C (t/ha)	<=10		11-25		26-50		51-75		76-100		>100	
	km^2	%	km^2	%	km^2	%	km^2	%	km^2	%	km^2	%
**Region**												
Central America	1,621	1	34,942	13	106,168	39	68,229	25	30,360	11	27,915	10
Central Asia	816,972	98	13,678	2	299	0	-	-	-	-	-	-
East Asia	1,066,727	59	438,840	24	255,198	14	34,410	2	718	0	-	-
Eastern and Southern Africa	881,523	56	534,088	34	96,050	6	37,796	2	16,558	1	7,512	0
Europe	1,809,606	79	406,180	18	83,223	4	757	0	-	-	-	-
North Africa	135,905	87	19,941	13	102	0	-	-	-	-	-	-
North America	1,313,470	63	411,039	20	204,567	10	96,319	5	29,221	1	18,417	1
Oceana	476,741	60	102,461	13	73,334	9	48,337	6	22,538	3	67,247	9
Russia	1,596,327	96	69,913	4	2,915	0	11	0	-	-	-	-
South America	321,760	8	1,964,145	51	1,028,356	26	334,189	9	124,305	3	116,037	3
South Asia	1,086,309	59	588,574	32	109,596	6	22,977	1	10,740	1	8,829	0
SouthEast Asia	226,738	14	266,556	16	292,212	18	249,594	15	186,845	11	426,323	26
West and Central Africa	1,573,021	66	255,654	11	217,659	9	151,654	6	72,708	3	120,284	5
Western Asia	842,890	88	76,679	8	31,139	3	4,948	1	33	0	-	-
**Global Total**	12,149,610	55	5,182,690	23	2,500,818	11	1,049,221	5	494,026	2	792,564	4

Figure S4: Carbon biomass (tC/ha) on agricultural land by Aridity-Wetness Index (AWI) in the year 2000 and 2010, globally and by global regions (Higher AWI values indicate more humid bioclimatic conditions).

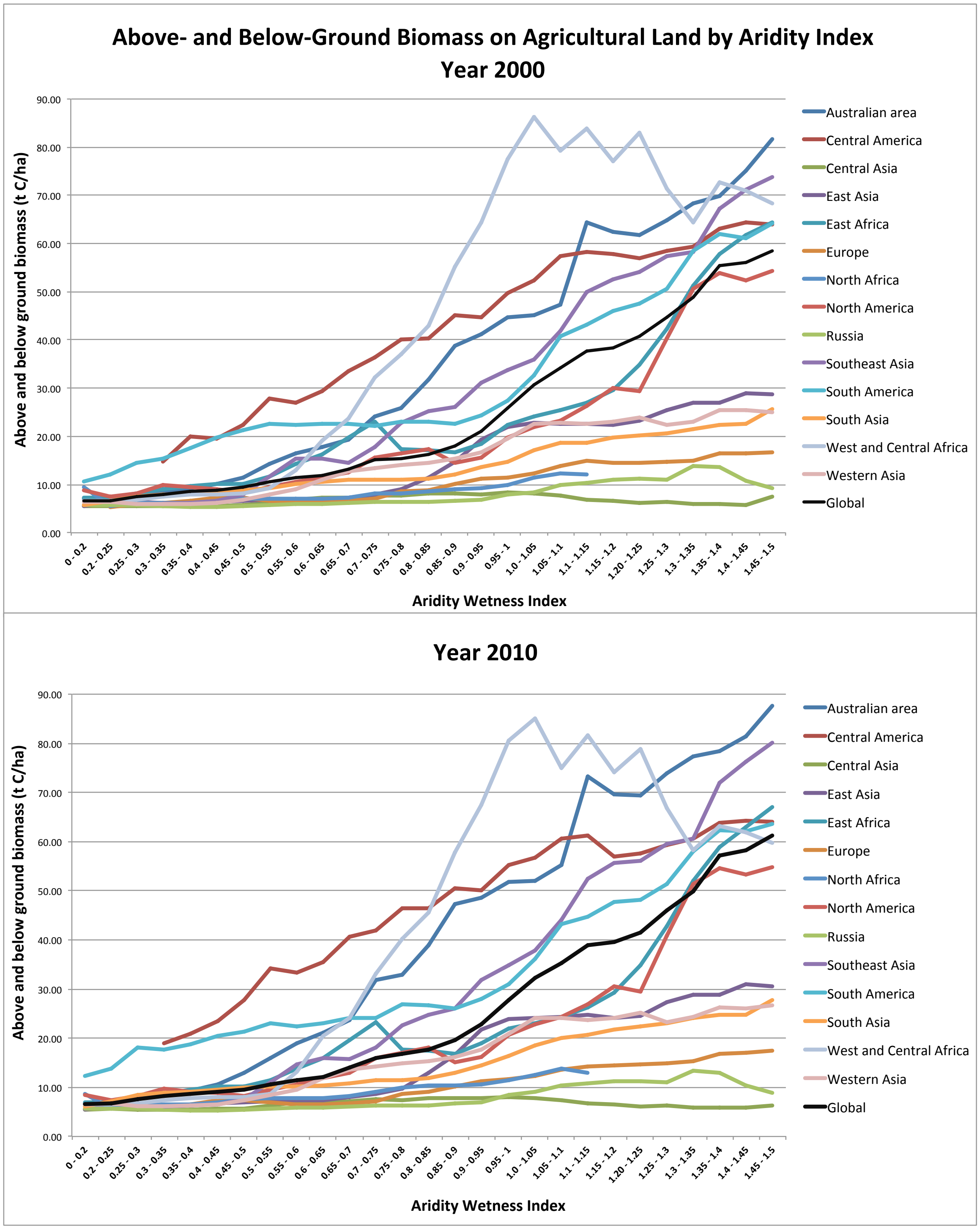

Figure S5: Average biomass carbon per hectare on agricultural land (tC ha-1) in 2000 and 2010, and the change in average biomass carbon from 2000 to 2010. Maps were produced based upon a spatial analysis using ESRI ArcGIS software (version 10.3; http://www.esri.com/software/arcgis/arcgis-for-desktop).

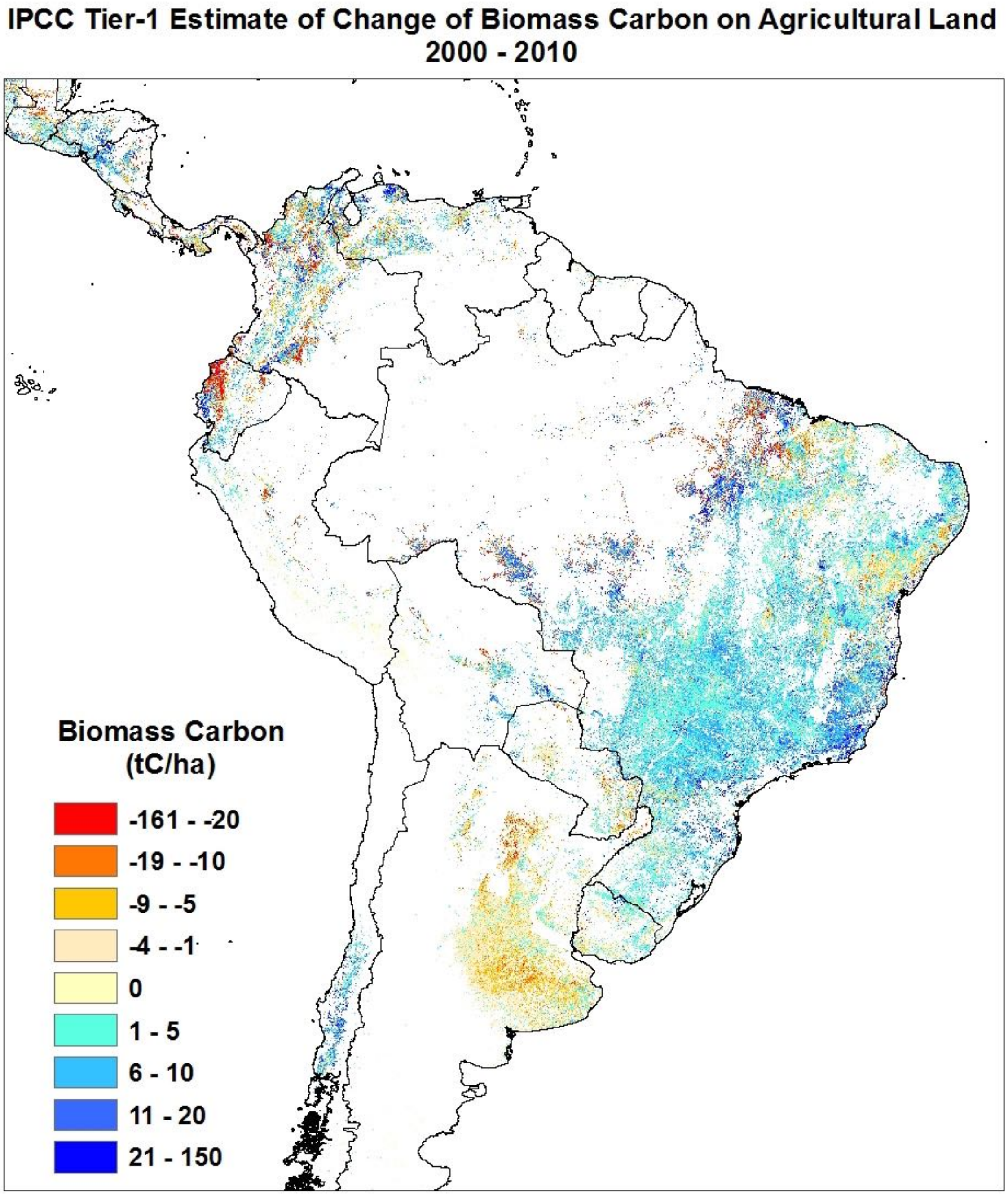

Figure S6: Average biomass carbon per hectare on agricultural land (tC ha-1) in 2000 and 2010, and the change in average biomass carbon from 2000 to 2010. Maps were produced based upon a spatial analysis using ESRI ArcGIS software (version 10.3; http://www.esri.com/software/arcgis/arcgis-for-desktop).

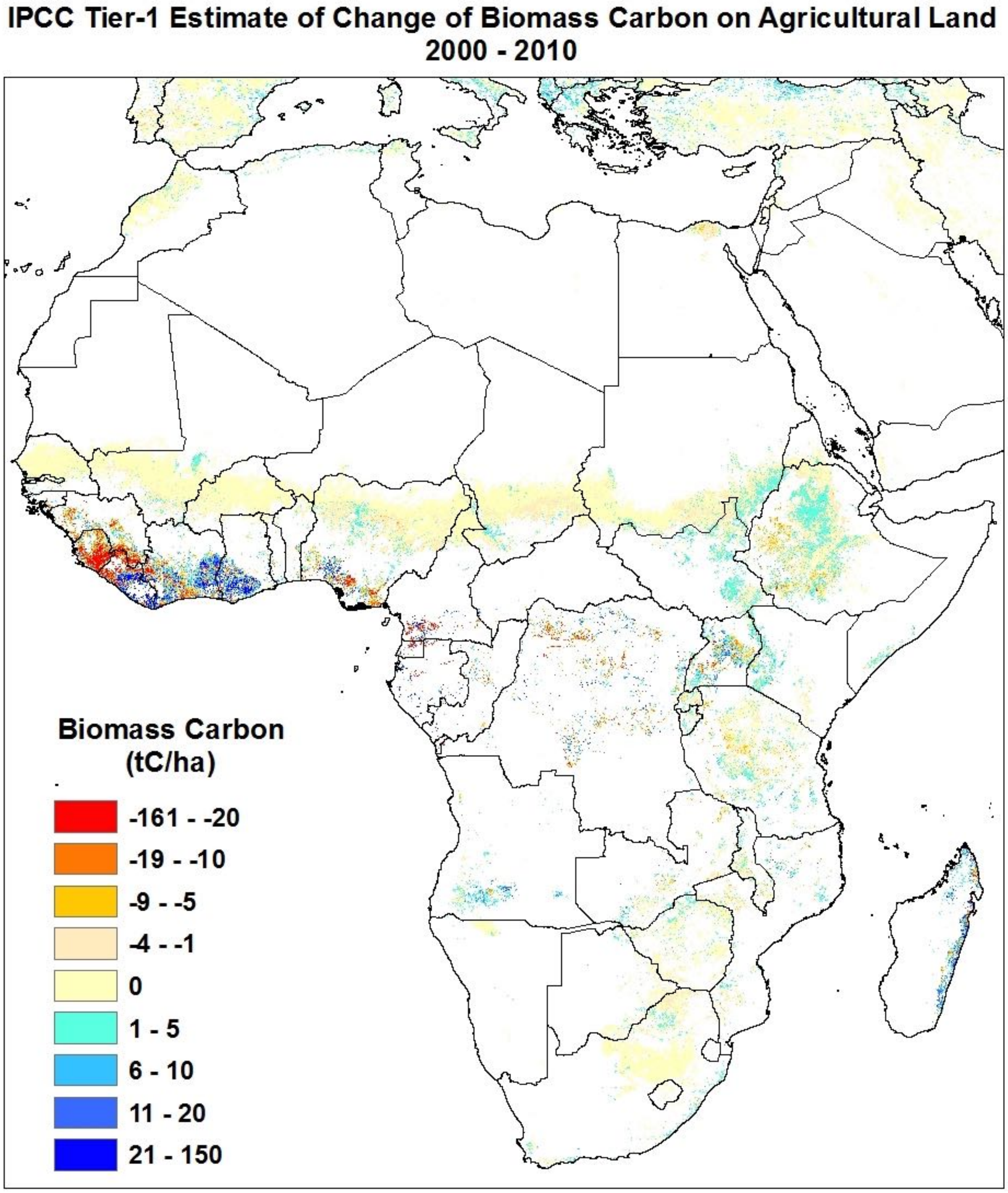

Figure S7: Average biomass carbon per hectare on agricultural land (tC ha^{-1}) in 2000 and 2010, and the change in average biomass carbon from 2000 to 2010. Maps were produced based upon a spatial analysis using ESRI ArcGIS software (version 10.3; http://www.esri.com/software/arcgis/arcgis-for-desktop).

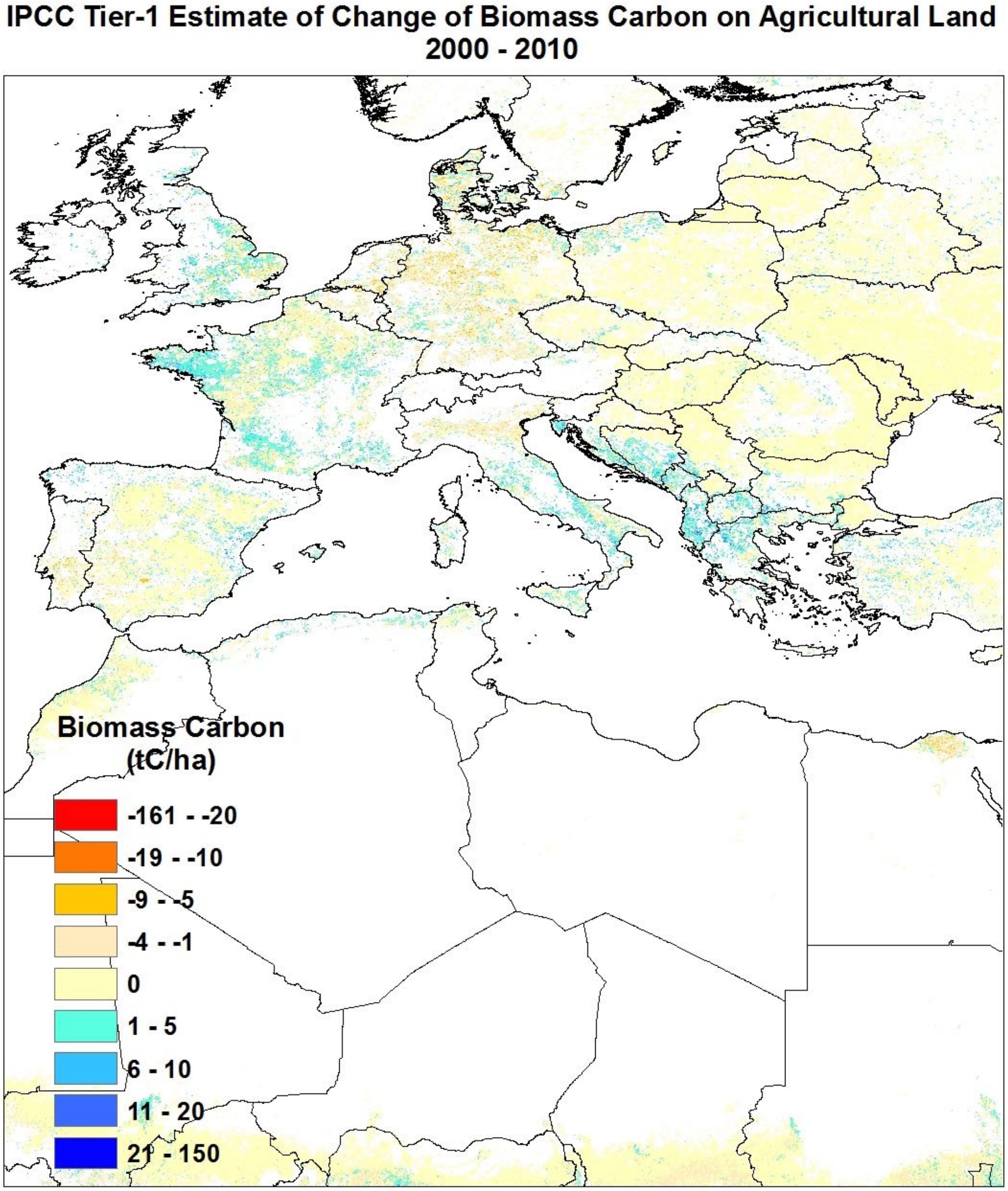

Figure S8: Average biomass carbon per hectare on agricultural land (tC ha^{-1}) in 2000 and 2010, and the change in average biomass carbon from 2000 to 2010. Maps were produced based upon a spatial analysis using ESRI ArcGIS software (version 10.3; http://www.esri.com/software/arcgis/arcgis-for-desktop).

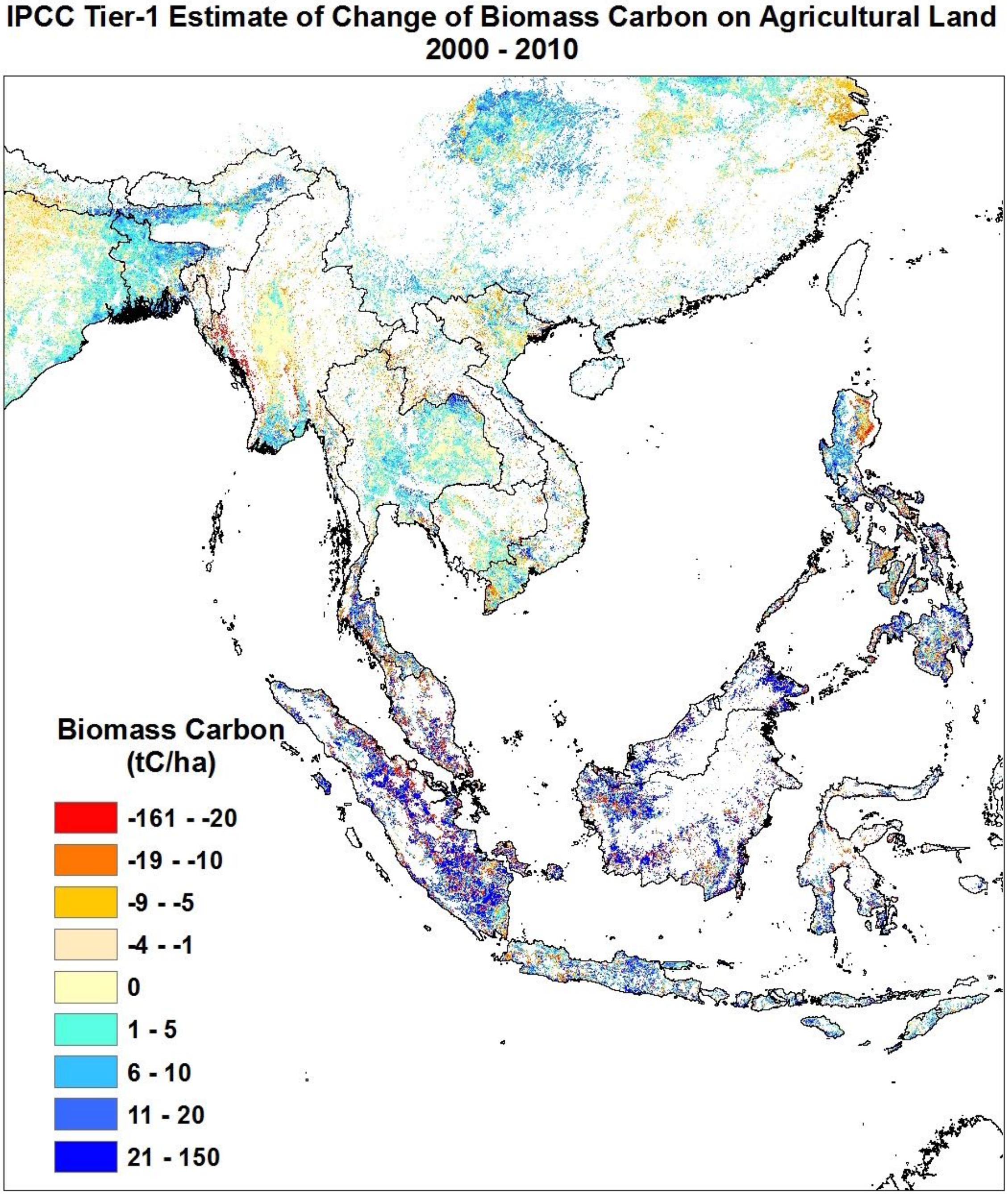

Figure S9: Average biomass carbon per hectare on agricultural land (tC ha^{-1}) in 2000 and 2010, and the change in average biomass carbon from 2000 to 2010. Maps were produced based upon a spatial analysis using ESRI ArcGIS software (version 10.3; http://www.esri.com/software/arcgis/arcgis-for-desktop).

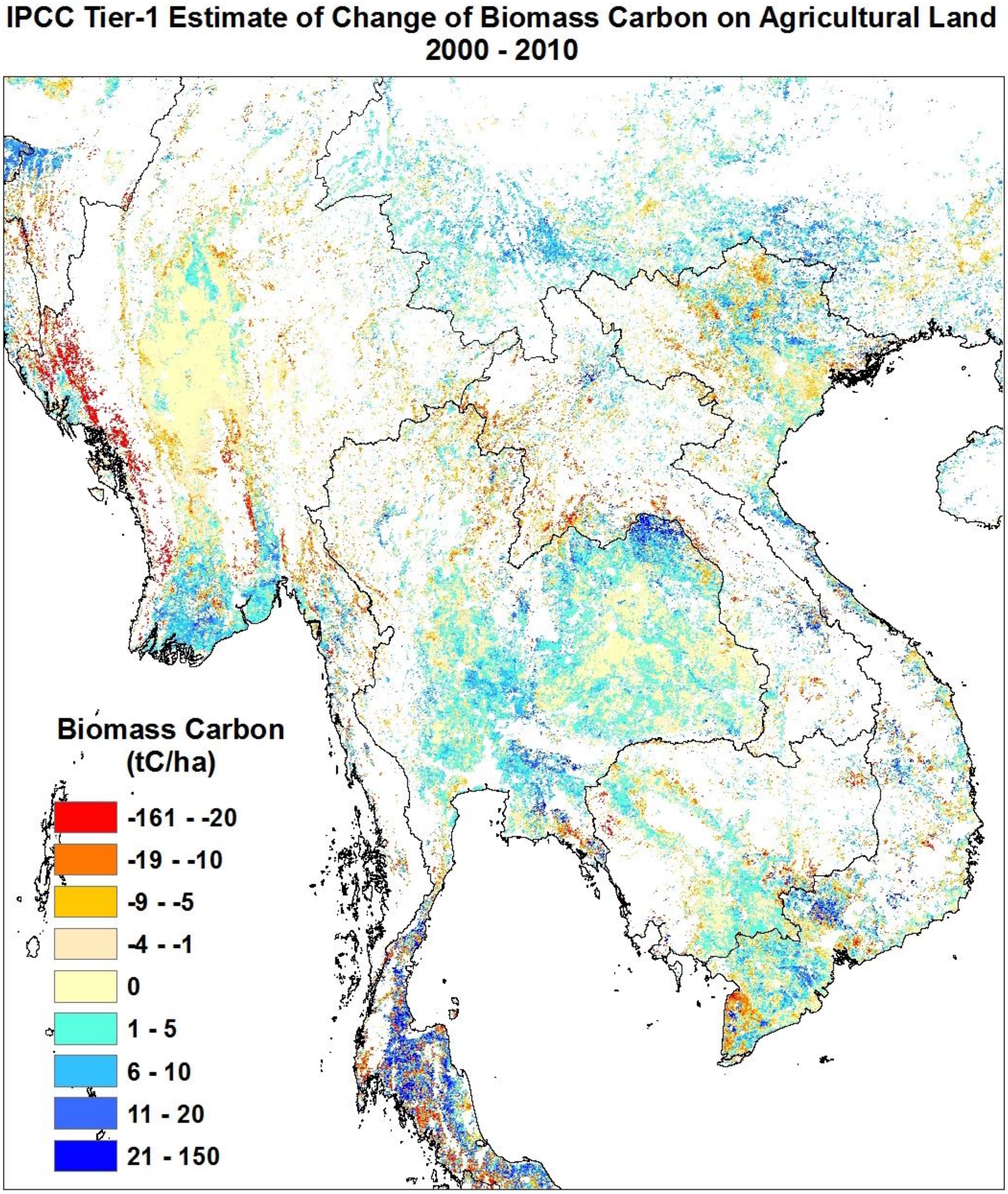

Figure S10: Average biomass carbon per hectare on agricultural land (tC ha^{-1}) in 2000 and 2010, and the change in average biomass carbon from 2000 to 2010. Maps were produced based upon a spatial analysis using ESRI ArcGIS software (version 10.3; http://www.esri.com/software/arcgis/arcgis-for-desktop).

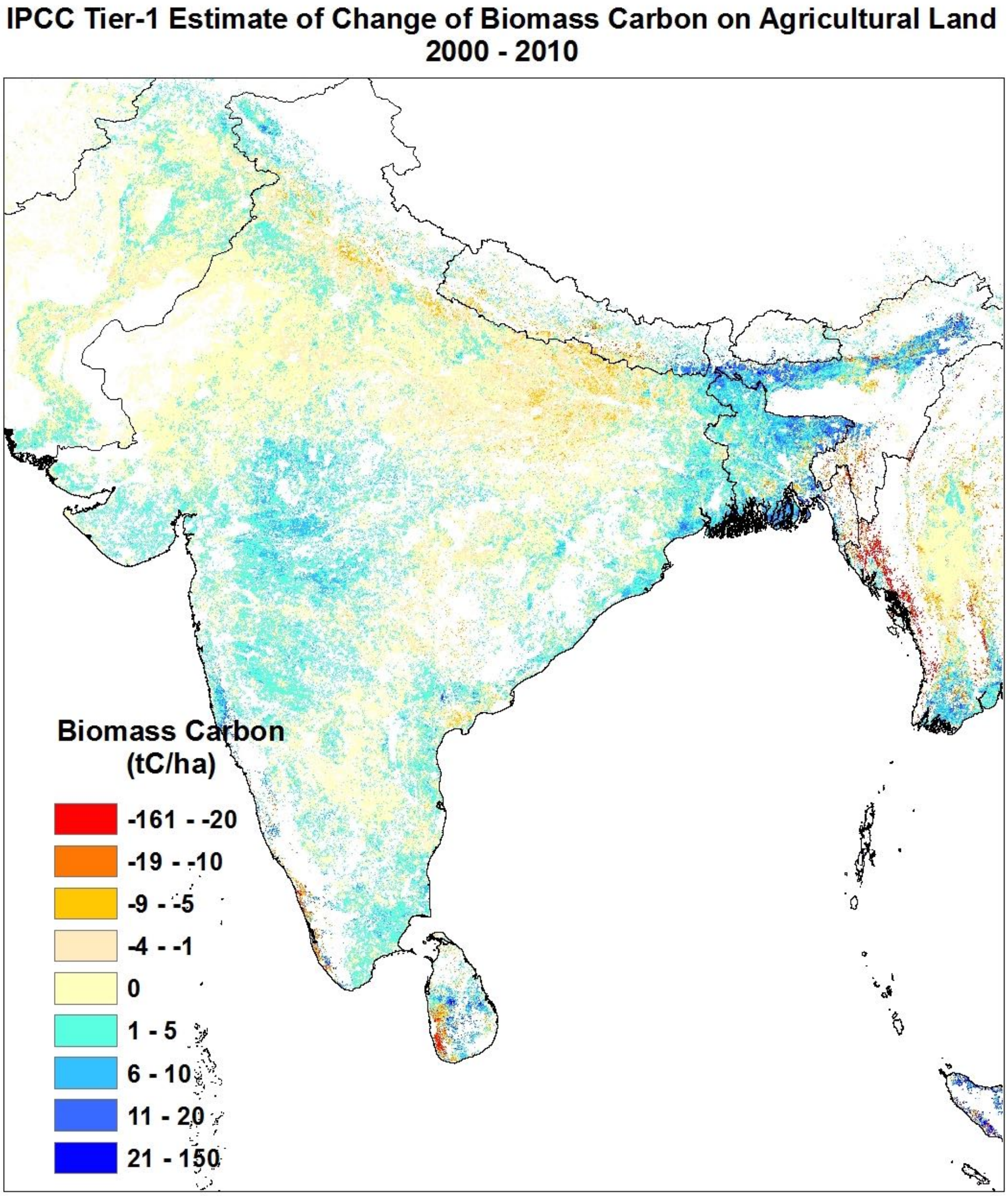

Figure S11: Average biomass carbon per hectare on agricultural land (tC ha^{-1}) in 2000 and 2010, and the change in average biomass carbon from 2000 to 2010. Maps were produced based upon a spatial analysis using ESRI ArcGIS software (version 10.3; http://www.esri.com/software/arcgis/arcgis-for-desktop).

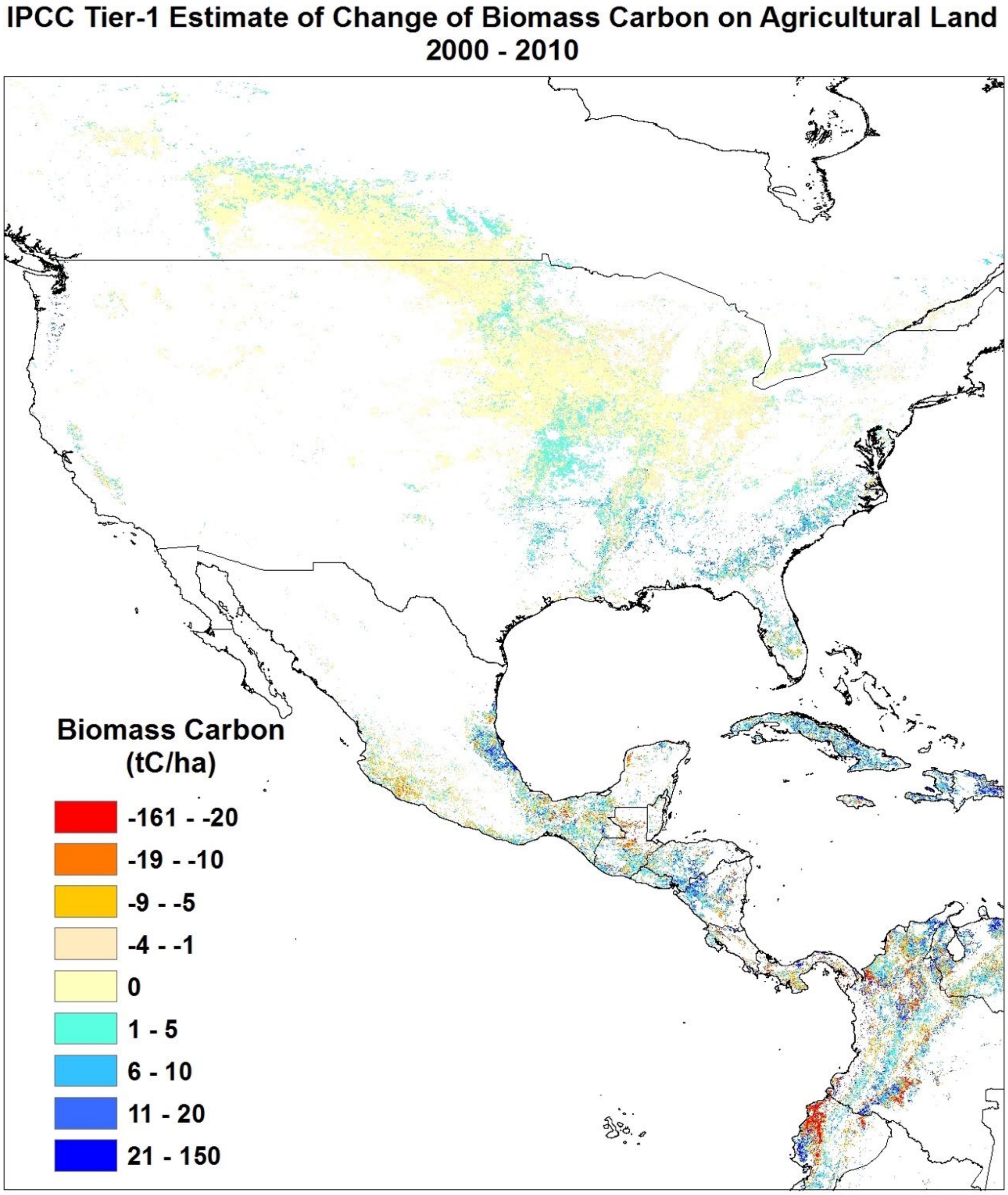

Table S1: Total biomass carbon on agricultural land (in PgC; and as a percentage of the total biomass carbon in 2000), and average per hectare biomass carbon (tC/ha), in the year 2000 and 2010 by country, grouped by region.

		Total Biomass Carbon (million t C)				Average Biomass Carbon			Total Agricultural Area (km²)
		t C * 10^6			Increase as % of Total	t C / ha			
Region	Country	2000	2010	Change		2000	2010	Change	
Australia / Pacific									
	Australia	595	635	39.9	6.7	10.0	10.6	0.7	597,768
	Fiji	23	26	3.5	15.4	81.4	94.0	12.5	2,814
	New Caledonia	31	35	3.9	12.4	59.3	66.6	7.4	5,273
	New Zealand	309	381	72.1	23.3	40.2	49.6	9.4	76,785
	Papua New Guinea	1094	1140	46.0	4.2	105.6	110.0	4.4	103,583
	Vanuatu	56	60	4.7	8.5	141.5	153.6	12.0	3,929
Central America									
	Belize	28	28	0.0	-0.1	74.1	74.0	-0.1	3,729
	Costa Rica	77	77	0.7	0.9	65.0	65.6	0.6	11,779
	Cuba	268	302	34.0	12.7	43.7	49.3	5.6	61,207
	Dominican Republic	114	131	17.5	15.4	63.4	73.2	9.8	17,945
	El Salvador	44	47	2.9	6.6	45.0	48.0	3.0	9,745
	Guatemala	262	256	-6.3	-2.4	63.3	61.8	-1.5	41,401
	Haiti	57	69	11.7	20.6	40.7	49.1	8.4	14,003
	Honduras	184	202	17.8	9.6	50.5	55.4	4.9	36,444
	Jamaica	40	41	1.1	2.9	86.8	89.3	2.5	4,564
	Nicaragua	191	211	20.1	10.5	48.5	53.6	5.1	39,408
	Panama	144	134	-9.6	-6.7	53.2	49.6	-3.6	26,989
	Puerto Rico	12	14	1.9	16.4	88.0	102.5	14.4	1,343
Central Asia									
	Kazakhstan	270	265	-4.6	-1.7	5.5	5.4	-0.1	492,142
	Kyrgyzstan	6	6	-0.2	-2.8	6.8	6.6	-0.2	8,872
	Mongolia	151	151	0.0	0.0	6.2	6.2	0.0	244,080
	Tajikistan	4	4	0.0	-0.9	5.9	5.9	-0.1	6,012
	Uzbekistan	32	32	-0.1	-0.4	6.0	6.0	-0.0	53,086
East Asia									
	China	2153	2320	166.4	7.7	12.7	13.7	1.0	1,692,194
	Japan	157	153	-3.2	-2.0	32.6	32.0	-0.7	47,997
	North Korea	14	14	0.0	-0.0	6.2	6.2	-0.0	22,659
	South Korea	38	39	0.8	2.0	12.3	12.6	0.2	31,124
	Taiwan	1	2	0.2	15.8	16.0	18.5	2.5	822
Eastern and Southern Africa									
	Angola	74	83	9.3	12.6	14.6	16.4	1.8	50,701
	Botswana	34	33	-0.8	-2.5	7.2	7.1	-0.2	46,490
	Burundi	15	14	-0.7	-4.5	16.0	15.2	-0.7	9,291
	Eritrea	17	17	-0.7	-4.0	7.6	7.3	-0.3	22,580
	Ethiopia	584	564	-19.8	-3.4	13.4	13.0	-0.5	435,230
	Kenya	80	85	4.5	5.7	13.6	14.4	0.8	59,217
	Lesotho	3	3	-0.1	-1.8	6.5	6.3	-0.1	4,503
	Madagascar	396	420	24.1	6.1	59.8	63.4	3.6	66,178
	Malawi	39	37	-1.3	-3.5	12.3	11.9	-0.4	31,219
	Mozambique	109	105	-4.0	-3.6	15.3	14.7	-0.6	71,335
	Namibia	9	10	0.4	4.6	5.1	5.4	0.2	17,929
	Rwanda	17	16	-1.1	-6.3	13.4	12.5	-0.8	12,938
	Somalia	12	13	0.9	7.2	9.8	10.5	0.7	12,336
	South Africa	173	174	0.5	0.3	7.3	7.3	0.0	237,806
	Tanzania	264	248	-15.9	-6.0	11.6	10.9	-0.7	226,692
	Uganda	297	302	4.8	1.6	32.8	33.3	0.5	90,560
	Zambia	89	85	-4.0	-4.4	14.2	13.6	-0.6	62,813
	Zimbabwe	90	90	-0.5	-0.5	7.9	7.9	-0.0	114,048

Table S1 (continued): Total biomass carbon on agricultural land (in PgC; and as a percentage of the total biomass carbon in 2000), and average per hectare biomass carbon (tC/ha), in the year 2000 and 2010 by country, grouped by region.

		Total Biomass Carbon (million t C)				Average Biomass Carbon			Total Agricultural Area (km^2)
		t C * 10^6			Increase as % of Total	t C / ha			
Region	Country	2000	2010	Change		2000	2010	Change	
Eurasia (Russia)									
	Russia	1067	1067	0.2	0.0	6.4	6.4	0.0	1,668,977
Europe									
	Albania	16	20	4.5	28.6	10.9	14.0	3.1	14,489
	Austria	12	12	0.1	0.8	9.4	9.5	0.1	12,829
	Belarus	71	74	2.4	3.4	6.6	6.8	0.2	108,507
	Belgium	17	15	-1.4	-8.5	16.1	14.7	-1.4	10,247
	Bosnia and Herzegovina	39	43	3.2	8.2	13.6	14.7	1.1	28,947
	Bulgaria	40	43	3.0	7.4	6.4	6.9	0.5	62,433
	Croatia	27	29	1.8	6.8	10.2	10.9	0.7	26,571
	Czech Republic	31	31	-0.3	-1.0	8.0	8.0	-0.1	38,496
	Denmark	44	42	-2.2	-5.0	15.8	15.0	-0.8	27,700
	Estonia	13	13	-0.1	-0.7	7.7	7.7	-0.1	17,398
	Finland	23	22	-1.0	-4.5	13.3	12.7	-0.6	17,083
	France	335	349	13.8	4.1	13.5	14.1	0.6	248,310
	Germany	280	256	-23.5	-8.4	16.5	15.1	-1.4	169,860
	Greece	36	43	6.9	19.1	9.6	11.4	1.8	37,540
	Hungary	39	41	1.5	3.9	5.9	6.1	0.2	66,719
	Ireland	4	5	0.5	11.1	18.0	20.0	2.0	2,295
	Italy	112	115	3.3	2.9	12.1	12.5	0.4	92,263
	Kosovo	5	6	0.4	8.2	7.5	8.1	0.6	7,217
	Latvia	21	21	-0.2	-0.7	7.4	7.4	-0.1	28,738
	Lithuania	26	26	0.1	0.4	6.7	6.7	0.0	38,410
	Luxembourg	1	1	0.0	-3.2	21.3	20.7	-0.7	640
	Macedonia	10	12	2.3	22.9	8.8	10.8	2.0	11,555
	Moldova	18	18	-0.1	-0.6	5.9	5.8	-0.0	30,083
	Montenegro	13	14	0.9	7.1	18.9	20.3	1.3	7,005
	Netherlands	26	23	-2.9	-11.3	18.8	16.7	-2.1	13,788
	Norway	15	14	-0.5	-3.3	9.1	8.8	-0.3	16,200
	Poland	133	135	1.7	1.2	7.7	7.8	0.1	173,199
	Portugal	24	22	-2.6	-10.7	10.8	9.6	-1.1	22,727
	Romania	83	85	2.4	2.9	6.1	6.3	0.2	135,482
	Serbia	35	37	1.7	4.8	6.9	7.2	0.3	50,792
	Slovakia	13	13	0.3	2.5	7.1	7.3	0.2	18,168
	Slovenia	4	4	-0.1	-3.1	11.7	11.3	-0.4	3,341
	Spain	170	173	3.8	2.2	7.6	7.8	0.2	221,765
	Sweden	32	32	-0.8	-2.6	11.7	11.4	-0.3	27,783
	Switzerland	3	3	-0.2	-6.3	16.8	15.8	-1.1	1,686
	Ukraine	274	272	-2.2	-0.8	6.1	6.0	-0.0	451,254
	United Kingdom	86	90	3.9	4.5	14.8	15.5	0.7	58,019
North Africa									
	Algeria	12	14	2.0	16.2	7.0	8.1	1.1	17,609
	Egypt	30	26	-4.0	-13.3	14.8	12.8	-2.0	20,207
	Libya	2	2	0.0	-1.2	5.2	5.2	-0.1	3,000
	Mauritania	15	15	0.0	-0.3	5.7	5.7	-0.0	26,831
	Morocco	49	50	1.4	2.9	6.3	6.5	0.2	77,767
	Tunisia	6	7	0.6	9.5	6.1	6.6	0.6	10,487
North America									
	Canada	333	346	12.7	3.8	7.6	7.9	0.3	439,934
	Mexico	1251	1250	-0.8	-0.1	47.0	47.0	-0.0	266,087
	United States	1727	1804	76.8	4.4	12.6	13.2	0.6	1,366,923

Table S1 (continued): Total biomass carbon on agricultural land (in PgC; and as a percentage of the total biomass carbon in 2000), and average per hectare biomass carbon (tC/ha), in the year 2000 and 2010 by country, grouped by region.

		Total Biomass Carbon (million t C)				Average Biomass Carbon			Total
		t C * 10^6			Increase as %	t C / ha			Agricultural
Region	Country	2000	2010	Change	of Total	2000	2010	Change	Area (km^2)
South America									
	Argentina	874	699	-175.0	-20.0	17.8	14.2	-3.6	491,951
	Bolivia	284	297	12.5	4.4	40.1	41.9	1.8	70,932
	Brazil	6790	7729	938.8	13.8	26.8	30.5	3.7	2,535,884
	Chile	153	189	35.7	23.4	31.2	38.5	7.3	49,040
	Colombia	1499	1493	-5.8	-0.4	52.7	52.4	-0.2	284,672
	Ecuador	413	376	-37.6	-9.1	50.6	46.0	-4.6	81,712
	French Guiana	5	6	0.9	18.3	83.1	98.3	15.2	599
	Guyana	18	18	0.2	1.2	50.1	50.7	0.6	3,541
	Paraguay	235	221	-14.2	-6.0	29.1	27.4	-1.8	80,714
	Peru	278	278	-0.9	-0.3	37.5	37.4	-0.1	74,301
	Suriname	13	14	0.3	2.3	62.5	64.0	1.4	2,112
	Uruguay	129	134	4.1	3.2	19.8	20.4	0.6	65,346
	Venezuela	647	677	29.4	4.5	44.0	46.0	2.0	147,179
South Asia									
	Bangladesh	207	248	41.0	19.8	20.3	24.4	4.0	101,875
	Bhutan	28	30	1.8	6.5	43.8	46.7	2.9	6,394
	India	1834	1970	136.0	7.4	11.2	12.0	0.8	1,640,067
	Nepal	109	109	-0.4	-0.4	22.1	22.0	-0.1	49,422
	Sri Lanka	119	121	1.9	1.6	41.1	41.7	0.6	29,056
SouthEast Asia									
	Cambodia	102	96	-5.7	-5.6	18.2	17.2	-1.0	55,846
	East Timor	36	39	3.0	8.4	56.2	60.9	4.7	6,387
	Indonesia	5493	6007	513.9	9.4	88.3	96.6	8.3	621,762
	Laos	248	236	-11.8	-4.8	54.0	51.4	-2.6	45,916
	Malaysia	1034	1140	105.8	10.2	96.9	106.8	9.9	106,718
	Myanmar	553	496	-57.2	-10.3	28.7	25.7	-3.0	193,138
	Philippines	1394	1458	64.7	4.6	73.7	77.1	3.4	189,123
	Thailand	696	738	41.9	6.0	25.3	26.8	1.5	275,437
	Vietnam	452	457	4.6	1.0	30.0	30.3	0.3	150,843
West and Central Africa									
	Benin	24	22	-1.3	-5.6	12.7	12.0	-0.7	18,576
	Burkina Faso	110	106	-3.6	-3.2	6.5	6.3	-0.2	168,316
	Cameroon	225	209	-16.6	-7.4	34.0	31.5	-2.5	66,206
	Central African Republic	15	14	-0.2	-1.5	14.0	13.8	-0.2	10,418
	Chad	191	179	-12.0	-6.3	7.4	6.9	-0.5	257,824
	Cote d'Ivoire	641	684	43.7	6.8	42.9	45.8	2.9	149,304
	D. R. Congo	1372	1350	-21.8	-1.6	113.0	111.2	-1.8	121,384
	Equatorial Guinea	40	33	-7.0	-17.6	93.8	77.3	-16.5	4,273
	Gabon	100	105	4.5	4.5	89.3	93.3	4.0	11,213
	Gambia	6	6	0.0	-0.8	9.0	9.0	-0.1	6,485
	Ghana	271	332	61.1	22.6	35.6	43.7	8.1	75,900
	Guinea	352	303	-49.0	-13.9	52.2	45.0	-7.3	67,377
	Guinea-Bissau	18	17	-0.6	-3.2	50.1	48.5	-1.6	3,607
	Liberia	443	442	-0.7	-0.1	78.6	78.5	-0.1	56,353
	Mali	166	163	-2.7	-1.6	7.0	6.8	-0.1	238,245
	Niger	15	15	-0.1	-0.4	5.9	5.9	-0.0	25,437
	Nigeria	545	513	-31.6	-5.8	12.8	12.1	-0.7	425,128
	Republic of Congo	134	138	3.8	2.9	96.9	99.7	2.8	13,830
	Senegal	74	72	-1.5	-2.0	6.6	6.5	-0.1	112,190
	Sierra Leone	370	279	-91.2	-24.7	67.7	51.0	-16.7	54,642
	South Sudan	163	171	8.6	5.3	11.3	11.9	0.6	144,182
	Sudan	284	281	-3.3	-1.2	8.1	8.0	-0.1	352,163
	Togo	13	13	-0.2	-1.7	19.9	19.5	-0.3	6,484

Table S1 (continued): Total biomass carbon on agricultural land (in PgC; and as a percentage of the total biomass carbon in 2000), and average per hectare biomass carbon (tC/ha), in the year 2000 and 2010 by country, grouped by region.

		Total Biomass Carbon (million t C)				Average Biomass Carbon			Total Agricultural Area (km^2)
		t C * 10^6			Increase as % of Total	t C / ha			
Region	Country	2000	2010	Change		2000	2010	Change	
Western Asia									
	Afghanistan	62	63	0.9	1.4	5.2	5.3	0.1	118,720
	Armenia	15	15	0.8	5.2	11.8	12.4	0.6	12,403
	Azerbaijan	31	32	0.2	0.7	9.5	9.5	0.1	33,220
	Cyprus	1	1	0.0	-1.5	6.2	6.1	-0.1	1,951
	Georgia	42	45	2.7	6.5	21.9	23.3	1.4	19,187
	Iran	81	82	0.9	1.1	7.0	7.1	0.1	115,378
	Iraq	28	28	0.1	0.3	5.3	5.3	0.0	52,831
	Israel	2	2	0.1	4.4	6.7	6.9	0.3	3,093
	Jordan	1	1	0.0	1.4	5.4	5.5	0.1	2,142
	Lebanon	1	1	0.1	5.9	6.8	7.2	0.4	1,720
	Pakistan	206	217	11.1	5.4	7.5	8.0	0.4	273,210
	Palestine	1	1	0.0	4.4	5.3	5.5	0.2	1,381
	Saudi Arabia	4	4	0.0	0.3	5.1	5.1	0.0	8,443
	Syria	8	9	0.2	2.1	6.2	6.3	0.1	13,677
	Turkey	263	282	18.5	7.0	9.0	9.6	0.6	291,990
	Turkmenistan	14	14	-0.1	-0.4	5.2	5.2	-0.0	26,874
	Yemen	3	3	0.0	0.7	5.9	5.9	0.0	5,538
